# Therapeutic Potential of Mesenchymal Stromal Cell-Derived Extracellular Vesicles in the Prevention of Organ Injuries Induced by Traumatic Hemorrhagic Shock

**DOI:** 10.3389/fimmu.2021.749659

**Published:** 2021-09-29

**Authors:** Guillaume Valade, Nicolas Libert, Christophe Martinaud, Eric Vicaut, Sébastien Banzet, Juliette Peltzer

**Affiliations:** ^1^ Institut de Recherche Biomédicale des Armées (IRBA), Inserm UMRS-MD-1197, Clamart, France; ^2^ Service d’Anesthésie-Réanimation, Hôpital d’instruction des armées Percy, Clamart, France; ^3^ Unité de Médicaments de Thérapie Innovante, Centre de Transfusion Sanguine des Armées, Clamart, France; ^4^ Laboratoire d’Etude de la Microcirculation, Université de Paris, UMRS 942 INSERM, Paris, France

**Keywords:** mesenchymal stromal cell, extracellular vesicles, inflammation, traumatic hemorrhagic shock, multi-organ failure, acute injury

## Abstract

Severe trauma is the principal cause of death among young people worldwide. Hemorrhagic shock is the leading cause of death after severe trauma. Traumatic hemorrhagic shock (THS) is a complex phenomenon associating an absolute hypovolemia secondary to a sudden and significant extravascular blood loss, tissue injury, and, eventually, hypoxemia. These phenomena are responsible of secondary injuries such as coagulopathy, endotheliopathy, microcirculation failure, inflammation, and immune activation. Collectively, these dysfunctions lead to secondary organ failures and multi-organ failure (MOF). The development of MOF after severe trauma is one of the leading causes of morbidity and mortality, where immunological dysfunction plays a central role. Damage-associated molecular patterns induce an early and exaggerated activation of innate immunity and a suppression of adaptive immunity. Severe complications are associated with a prolonged and dysregulated immune–inflammatory state. The current challenge in the management of THS patients is preventing organ injury, which currently has no etiological treatment available. Modulating the immune response is a potential therapeutic strategy for preventing the complications of THS. Mesenchymal stromal cells (MSCs) are multipotent cells found in a large number of adult tissues and used in clinical practice as therapeutic agents for immunomodulation and tissue repair. There is growing evidence that their efficiency is mainly attributed to the secretion of a wide range of bioactive molecules and extracellular vesicles (EVs). Indeed, different experimental studies revealed that MSC-derived EVs (MSC-EVs) could modulate local and systemic deleterious immune response. Therefore, these new cell-free therapeutic products, easily stored and available immediately, represent a tremendous opportunity in the emergency context of shock. In this review, the pathophysiological environment of THS and, in particular, the crosstalk between the immune system and organ function are described. The potential therapeutic benefits of MSCs or their EVs in treating THS are discussed based on the current knowledge. Understanding the key mechanisms of immune deregulation leading to organ damage is a crucial element in order to optimize the preparation of EVs and potentiate their therapeutic effect.

## 1 Introduction

Severe trauma is the main cause of death among young people worldwide (1, 2), one-third being attributed to hemorrhage (3). In the military population, during modern conflicts, 90% of preventable deaths are of hemorrhagic origin (4).

Hemorrhage secondary to trauma is an emergency that can evolve into traumatic hemorrhagic shock (THS). Hemorrhagic shock in such condition is a complex association of tissue injuries and a severe hypovolemia due to blood loss. This leads to circulatory failure and inadequate tissue perfusion that induces a switch from aerobic to anaerobic metabolism (5). This phenomenon is responsible for secondary insults with tissue damage and inflammation, which can progress in the worst cases to organ dysfunction and multi-organ failure (MOF). The incidence of MOF is high in cases of severe trauma and remains a major cause of morbidity and mortality (≈33%) (6, 7).

Severe trauma is most often accompanied by significant tissue damage. Tissue attrition will rapidly lead to significant inflammation. The current challenge in the management of THS patients is preventing organ injuries, which currently have no etiological treatment available. Indeed, whereas post-hemorrhage resuscitation improves tissue perfusion, it does not treat the complex mechanisms that occur with reperfusion (ischemia/reperfusion, I/R) and activation of inflammatory and immune responses. Inflammatory and immune burst after trauma are major contributors of MOF (8, 9). The immune cells then become adherent to the vascular wall and decrease distal blood flow. These phenomena then induce tissue hypoperfusion, responsible for dysfunction of the microcirculation, hypoxia, and cellular acidosis, rapidly leading to organ failure and MOF. To improve the prognosis of patients, there is a critical need for new therapies to prevent and treat organ dysfunction and MOF after trauma.

Modulation of the immune and inflammatory response is a promising therapeutic strategy to treat complications of THS.

Mesenchymal stromal cells (MSCs) were discovered in the 1970s. Alexander Friedenstein, demonstrated the ability of culture-isolated fibroblast cells (now designated as MSCs) to recreate a hematopoietic environment *in vivo* after heterotopic grafting (10). These pioneering experiments provided the first clues to the existence of a cellular memory of the function they exerted in their original tissue. MSCs in the medullary microenvironment participate in the regulation of self-renewal and differentiation of hematopoietic stem cells (HSCs). More recently, clinical trials have shown that the co-graft of MSCs and HSCs allowed for better engraftment of HSCs while decreasing the risk of graft *vs*. host disease (GvHD) (11–14). Since then, many studies have shown the immunomodulatory capacities of MSCs in different contexts *in vitro* and *in vivo* and notably after trauma (15–17). MSCs exert their immunomodulation capacities by cell-to-cell contact or paracrine pathway *via* the secretion of various types of anti-inflammatory molecules and extracellular vesicles (EVs) (18).

In this review, we discuss the therapeutic potential and rationale for the application of EV-enriched MSC secretome for the prevention of organ injuries in an emergency context of THS.

## 2 Traumatic Hemorrhagic Shock

### 2.1 Epidemiology

Hemorrhagic shock is responsible for 1.9 million deaths per year worldwide, 79% of which are caused by physical trauma (1). According to the World Health Organization, 5.8 million deaths per year are due to trauma, which represents 10% of the causes of death (19). The majority of deaths occur at the site of the trauma or in the first hours of medical management, mainly as a result of brain injury or circulatory collapse following hemorrhage. Hospital deaths are the result of sepsis or MOF (20, 21). In modern conflicts, blast injuries have become predominant and account for nearly 75% of combat casualties in Iraq and Afghanistan (22). These injuries mainly concern poorly protected areas (limbs and the head and neck axis) in 34% of cases (23). Among soldiers killed in action, 87% died before reaching a medical facility, 24% of these deaths being considered to be potentially preventable. More than 90% of these potentially preventable deaths are associated with hemorrhage (4). During the last decade, the strategy to decrease the mortality rate was to prevent pre-hospital exsanguination. This has been partially achieved by the large diffusion of massive bleeding control strategies based on compressive devices such as tourniquets (24). However, the time of the pre-hospital phase has been considerably increased in recent conflicts (Sahel), promoting the duration of the shock and the onset of complications (25, 26).

### 2.2 Pathophysiology

The pathophysiology of THS is complex. We describe this phenomenon from the clinical to the cellular aspect, then discuss the 2021 guidelines for the management of critically ill patients without comorbidity factors.

THS associates tissue trauma and hemorrhagic shock, a form of hypovolemic shock in which sudden and severe blood loss leads to inadequate oxygen delivery at the cellular level (5). Hypovolemia causes a drop in venous return, blood pressure, and stroke volume. The clinical manifestations of shock include tachycardia, tachypnea, sweat, pallor, oliguria, and confusion. The clinical definition of shock associates one or several of these signs to a systolic blood pressure <90 mmHg. Metabolic cell activity is strongly dependent on the oxygen supply (DO_2_). The dioxygen artery concentration (CaO_2_) depends first on O_2_ binding to hemoglobin (Hb) and dioxygen saturation (SaO_2_) and, second, on dissolved (PaO_2_) (27). During hemorrhage, DO_2_ decreases because of a drop in Hb, cardiac output, or SaO_2_. Because of this drop, aerobic cell metabolism switches from aerobic to anaerobic metabolism, allowing the cell to maintain a minimal energy production (cf. *Section 2.2.1*). To maintain a sufficient DO_2_, the number of perfused capillaries increases (i.e., capillary recruitment) in proportion to the degree of tissue hypoxia, the oxygen extraction ratio increases, and regional vascular resistance is lowered to induce blood flow redistribution (28).

The adaptive mechanisms allowing the adaptation of the organism are neurological, renal, and hormonal. These can lead to the three phases of THS: compensated, decompensated and exceeded (29).

#### 2.2.1 Compensated THS

In the compensated shock phase, tissue hypoperfusion is counterbalanced by adaptive mechanisms.

The decrease in blood pressure is quickly detected by cardiopulmonary and arterial baroreceptors that induce an increase in sympathetic activity, resulting in arteriolar and venous vasoconstriction and an increase in heart rate to preserve vital organs such as the heart, lungs, and brain (30, 31). The renin–angiotensin–aldosterone system is also activated. Angiotensin promotes a ubiquitous vasoconstriction and stimulates aldosterone and anti-diuretic hormone production, sympathetic heart stimulation, thirst sensation, and decreased glomerular filtration rate (GFR) (32). Altogether, these compensatory mechanisms maintain the cardiac output, perfusion pressure, and circulating volume. All cellular functions are maintained as long as the combined yields of the aerobic and anaerobic sources of energy provide sufficient ATP (28). Nevertheless, these compensations can be overwhelmed.

#### 2.2.2 Decompensated THS

When blood loss reaches a critical level (30%–40%) (29, 30), the compensatory mechanisms are overwhelmed: there is a massive decrease of reflex-activated sympathetic drive and an increase in cardiac vagal drive, resulting in reductions in heart rate and arterial blood pressure and loss of peripheral resistances (30). Uncompensated THS resulting in irreversible tissue damage occurs when the combined aerobic and anaerobic ATP supplies are not sufficient to maintain cellular function (28).

#### 2.2.3 Exceeded THS

This last phase is associated with a “no reflow,” even if volemia is restored. Neutrophils adhere to the damaged endothelium, block capillaries, and aggravate local ischemic injuries. This worsens lesions such as coagulopathy, endotheliopathy, microcirculation failure, inflammation, and immune activation. All of these lead to secondary organ failure, MOF, and death (29).

### 2.3 From Cellular Insults to MOF

#### 2.3.1 Cellular Insult Due to Ischemia/Reperfusion

The shift from aerobic to anaerobic metabolism results in the formation of lactate and protons and a decrease in ATP production. pH is maintained *via* H^+^/Na^+^ and Na^+^/Ca^2+^ pumps, causing an elevation of cytosolic Ca^2+^ (33). Moreover, ATP production is insufficient to maintain the function of these pumps. A disruption of the mitochondrial architecture also occurs, which destabilizes the mitochondrial membrane potential. This membrane potential is further affected by the opening of the mitochondrial permeability transition pore and inner membrane anion channels, finally impairing ATP production (34). The damaged mitochondria are no longer able to efficiently reduce O_2_ in H_2_O in the electron transport chain, leading to reactive oxygen species (ROS) formation (35). Oxidative stress is usually defined as an imbalance between the production of ROS and antioxidants. The ensuing pathophysiological consequences and oxidative damages correspond to protein nitrosylation, lipid peroxidation, or DNA damage and can lead to cell death. Necrotic cells and damage to the extracellular matrix release various intracellular and extracellular molecules, which act as “alarmins” triggering inflammatory cascades (36).

#### 2.3.2 Activation of Inflammation During THS

##### 2.3.2.1 Alarm Signals

“Alarmins,” among which damage-associated molecular patterns (DAMPs) are released with tissue injuries, trigger both an intense pro-inflammatory systemic immune response syndrome (SIRS) and a counterbalancing anti-inflammatory response syndrome (CARS) within 30 min post-injury (37). Every DAMP proven to induce efferent pro-inflammatory pathways can be involved in the development of SIRS (38). This highlights the critical role of DAMPs in SIRS-associated MOF following THS. Moreover, it has been recently described that suppressing inducible DAMPs (SAMPs) (39), mainly produced by activated leukocytes and macrophages upon stress and injury (e.g., lipid mediators such as prostaglandin E2 or annexin A1) (40, 41), could trigger the pro-resolving pathways in CARS. An excessive CARS could lead to posttraumatic immunosuppression. In this review, we mainly focus on the mechanisms of THS-induced SIRS. DAMPs are passively released by necrotic cells, but also actively secreted by stressed or activated cells (e.g., high mobility group box protein 1, HMGB1). Elevated levels of HMGB1 (42–46), mtDNA (47–52), heat shock proteins (53–57), Ca^2+^-binding protein S100 (58), histones (59–63), ATP (64), interleukin 33 (IL-33) (65), or IL-1 (66) have been described after trauma in preclinical and clinical studies. DAMPs activate immune cells *via* their binding to pattern recognition receptors (PRRs), a group of receptors involved in the innate immune response, and induce the transcription of inflammatory factors (67, 68). Toll-like receptors (TLRs) form the most prominent group (69), and Nod-like receptors (NLRs) such as NLRP3 (70), receptor for advanced glycation end products (RAGE) (71), and purinergic (72) or complement receptors (73) also contribute to inflammation. The activation of these receptors triggers multiple pathways, notably the tumor necrosis factor alpha (TNF-α)/nuclear factor kappa B (NF-κB)/c-Jun N-terminal kinase (JNK) and p38 mitogen-activated protein kinase (MAPK) signaling cascade (42, 66, 74–77), and the activation of NLRP3 inflammasome with production of IL-1β or IL-18 (78). In the case of major THS, the massive release of DAMPs may induce an excessive innate immune response, leading to coagulopathy, endothelial dysfunction, and an increase in vascular permeability, promoting the circulation of new DAMPs. This amplifies a vicious cycle of cell and tissue injuries that heightens the immunological response (73, 78). Resident inflammatory cells have the role of sentinels. They detect an increase in circulating DAMPs, and then they trigger the recruitment of circulating immune cells by releasing TNF-α, IL-6, IL-1β, etc. (34, 79, 80). DAMPs could also be secreted by activated immune cells such as neutrophils or monocytes and are also potent activators of the complement, leading to the generation of C3a, iC3b, and C5a (81, 82). Elevated plasma C3a, C5a, and C5b-9 levels correlate with trauma severity (83–85), and complement activation also contributes to neutrophil and monocyte recruitment (34).

##### 2.3.2.2 Granulocytes: In the First Line

Knowledge of the immune changes during the early phase is limited. A study on severe trauma patients revealed a massive leukocytosis, elevated serum pro- and anti-inflammatory cytokines, and evidence of innate cell activation within minutes of trauma (86).

The SIRS-primed circulating neutrophils home to the tissues and become activated by local inflammatory stimuli (87). Notably, data obtained in a cohort of trauma patients suggest that circulating platelet-activating factor (PAF) and IL-8 are potential mechanisms of circulating neutrophil priming. Indeed, the use of a PAF antagonist inhibits neutrophils priming 3 h after injury, and plasmatic levels of IL-8 increase between 6 and 12 h after injury. Moreover, at 12 h, IL-8 may also be an early predictive marker of the onset of MOF (88). Circulating neutrophil activation is associated with reduced surface expressions of CXCR2 (CD182) and C5aR (CD88) 3–4 h after injury, followed by gradual restoration (86, 89). Then, the expressions of CD62L (L-selectin) and CXCR1 (CD181) start decreasing at about 4–12 h (86), and CD62L remains low at 24 h (90). These phenotypic changes are directly related with inflammation (87) and phagocytosis (91). C5aR promotes phagocytosis, and its expression is downregulated by the binding of C5a (92). Conversely, the expression of CD11b is increased (93). Traumatic injury also leads to marked alterations in the phenotype, function, and life span of circulating neutrophils (94–96).

Circulating neutrophil counts increased sharply 3 h after injury and then decreased within 12 h, suggesting end organ sequestration. The drop in circulating neutrophils was significantly greater in MOF than that in non-MOF patients (93). Neutrophils reach the damaged tissues by diapedesis in the post-capillary venules. Neutrophil binding to the endothelium is first controlled by selectins (CD62L that binds to CD62E and CD62P), which promote the initial rolling or tethering. Then, integrins (the β2 integrins CD11a and CD11b) induce firm adhesion. Examination of autopsy specimens from patients with MOF revealed the presence of neutrophils that varies from renal blood vessels to large-scale tissue infiltration of the lung (97). Neutrophil apoptosis was profoundly delayed in severely injured patients, as well as their tissue clearance, correlating with a high risk of MOF (98, 99). When neutrophils are exposed to pro-inflammatory signals, they release not only ROS and proteases but also neutrophil extracellular traps (NETs), which induce injuries in healthy tissues. During NETosis, neutrophils release decondensed chromatin and proteins including neutrophil elastase, cathepsin G, and myeloperoxidase (MPO), as well as histones in NETs (100–102), which participate in the pathophysiology of trauma (103). The level of circulating cell-free DNA (used as a marker of NET formation) is higher in SIRS trauma patients than that in healthy subjects (104, 105).

##### 2.3.2.3 Antigen-Presenting Cells: Pivot of the Inflammatory Reaction

**Figure 1:**
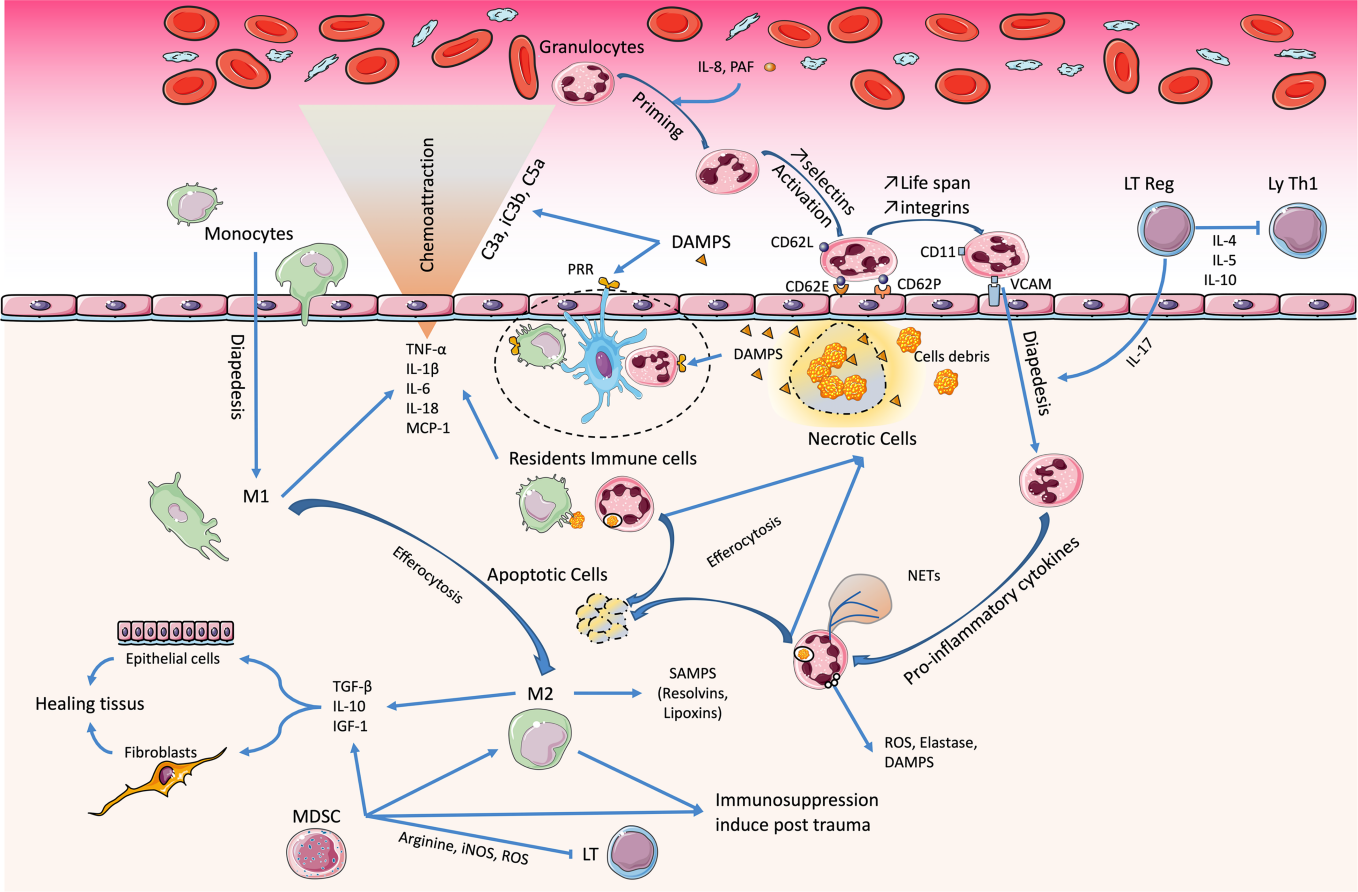
Immunological imbalance during traumatic hemorrhagic shock (THS). Damage-associated molecular patterns (DAMPs) play a key role in pathophysiology. The resident immune cells detect them and carry out the first reactions of phagocytosis and amplification of the inflammatory response. The circulating granulocytes infiltrate the tissue and maintain this reaction. Later-onset macrophages are pivotal in resolving this inflammatory phase (M1→M2) and initiate the healing phase. However, during THS, the abundance of DAMPs promotes the acquisition and maintenance of a pro-inflammatory phenotype. The trio of Treg, platelets, and endothelial cells co-stimulates and causes immunomodulation, with inhibition of Th1 lymphocytes. Bone marrow dysfunction induces an immunosuppression that favors the occurrence of sepsis.

Antigen-presenting cells (APCs), such as dendritic cells (DCs) and monocytes/macrophages, are important effector cells whose functional capacities are deeply influenced during tissue-induced injury (**Figure 1**). After THS, resident inflammatory cells serve as sentinels, then circulating neutrophil recruitment is rapidly followed by monocytes and macrophages. DAMPs bind to macrophage PRRs, leading to their pro-inflammatory activation, and can also trigger inflammasome formation, which does not support any direct transcriptional activity but allows the caspase-1-dependent cleavage of pro-IL-1β and pro-IL-18 into mature forms (37). It was recently demonstrated that inflammasomes, like TLRs, could trigger innate immune responses to aggression.

Functional phenotypical changes of macrophages from pro-inflammatory (M1) to anti-inflammatory (M2) occur to support tissue repair at the damaged sites. The clearance of neutrophils in tissues by efferocytosis represents a central element in the induction of the M1-to-M2 switch (106). These M2 macrophages secrete growth factors and anti-inflammatory cytokines such as IL-10, transforming growth factor beta (TGF-β), and IGF-1, which enhance tissue remodeling (79, 107) mediators of resolution (e.g., lipoxins and resolvins) (108) and increase their expression of the receptors programmed cell death ligands 1 (PD-L1) and 2 (PD-L2) (109, 110). Within 2–4 h after injury, the activation of the p38 MAPK, ERK1/2, and JNK pathways triggers macrophage activation in the liver, which releases TNF-α, IL-6, and remarkably high levels of monocyte chemoattractant protein-1 (MCP-1) and keratinocyte-derived chemokine. Macrophages are the major producers of MCP-1 and IL-6 after trauma–hemorrhage and contribute, at least in part, to the trauma/hemorrhage-associated neutrophil infiltration (111, 112).

As observed in sepsis, suppression of the function of monocytes/macrophages is directly associated with the severity of trauma (113). SIRS and CARS occur concomitantly, but when the CARS is excessive or persistent, it promotes immunosuppression, secondary infections, and late or persisting organ dysfunctions (114). Macrophage dysfunction is a significant contributor to both innate and adaptive immune suppression (115). This suppressive function is related to a decrease in human leukocyte antigen DR (HLA-DR) and CD86 expression (116, 117). This impairment in the antigen presentation of macrophages appears early after injury and is maintained for several days (118–121). In addition, DCs, which represent the most potent APCs for the induction of primary T-cell responses, show a reduced responsiveness to bacterial components within a few hours after trauma–hemorrhage, secrete reduced levels of TNF-α and IL-6, as well as INF-γ, IL12, and IL-12p40, and are less potent to induce T-cell proliferation (122).

##### 2.3.2.4 Bone Marrow Dysfunction

Maintaining the immune response following trauma requires the mobilization of bone marrow progenitors.

However, the formation of bone marrow granulocyte–macrophage colony-forming units (CFU-GM), erythroid burst-forming units (BFU-E), and erythrocyte colony-forming units (CFU-E) was significantly reduced, while peripheral blood CFU-GM, BFU-E, and CFU-E were increased in trauma patients. Bone marrow stroma failed to grow to confluence by day 14 in >90% of trauma patients. These data indicate that trauma induces a bone marrow dysfunction that releases immature white blood cells into circulation and may also contribute to a failure to clear infection and an increased propensity to organ failure (123, 124). Moreover, in pathophysiological conditions such as trauma, a partial blockade in the differentiation of immature myeloid cells into mature myeloid cells results in an expansion of this population called myeloid-derived suppressor cells (MDSCs), which have remarkable ability to suppress T-cell responses and to modulate macrophage cytokines (125). Moreover, MDSCs, like all APCs, interact and modulate the behavior of the adaptive immune system, notably T helper (Th) lymphocytes *via* major histocompatibility complex class II (MHCII), CD40, CD80, or CD86. MDSCs express low concentrations of MHCII and CD80/CD86 (126). The expansion of MDSC populations is proportional to the severity of the inflammatory insult (126, 127). Therefore, MDSCs could contribute to the post-trauma immunosuppression leading to the development of late sepsis and MOF (128).

##### 2.3.2.5 Adaptive Immune Response

The persistence of high levels of pro- and anti-inflammatory cytokines promotes T-cell exhaustion. There is a progressive decrease in the ability of T cells to produce cytokines (IFN-γ and TNF-α), higher expressions of CD28 and PD1 on CD4^+^ and a lower expression of CD127 on T cells, a loss of proliferative capacity, and a decreased cytotoxicity, which can lead to apoptotic cell death (129). Lymphocyte apoptosis occurs early after severe trauma and usually peaks at day 3 after the injury. There is a correlation between the injury severity score (ISS) and lymphopenia, aggravating the risk of subsequent major infection and sepsis (130). Apoptosis affects more the CD4^+^ and natural killer (NK) T lymphocytes than the CD8^+^. In contrast, the CD4^+^/CD25^+^ lymphocyte populations, regulatory T cells (Tregs), are more resistant to sepsis or burn-induced apoptosis (129, 131). Tregs are important mediators of the suppression of T-cell activation and the reduction in Th1 cytokine production after injury (132). Tregs also play a role in regulating neutrophils during I/R by modulating, for example, their sequestration diapedesis (133).

##### 2.3.2.6 Imbalance of Immunological Response

**Figure 2:**
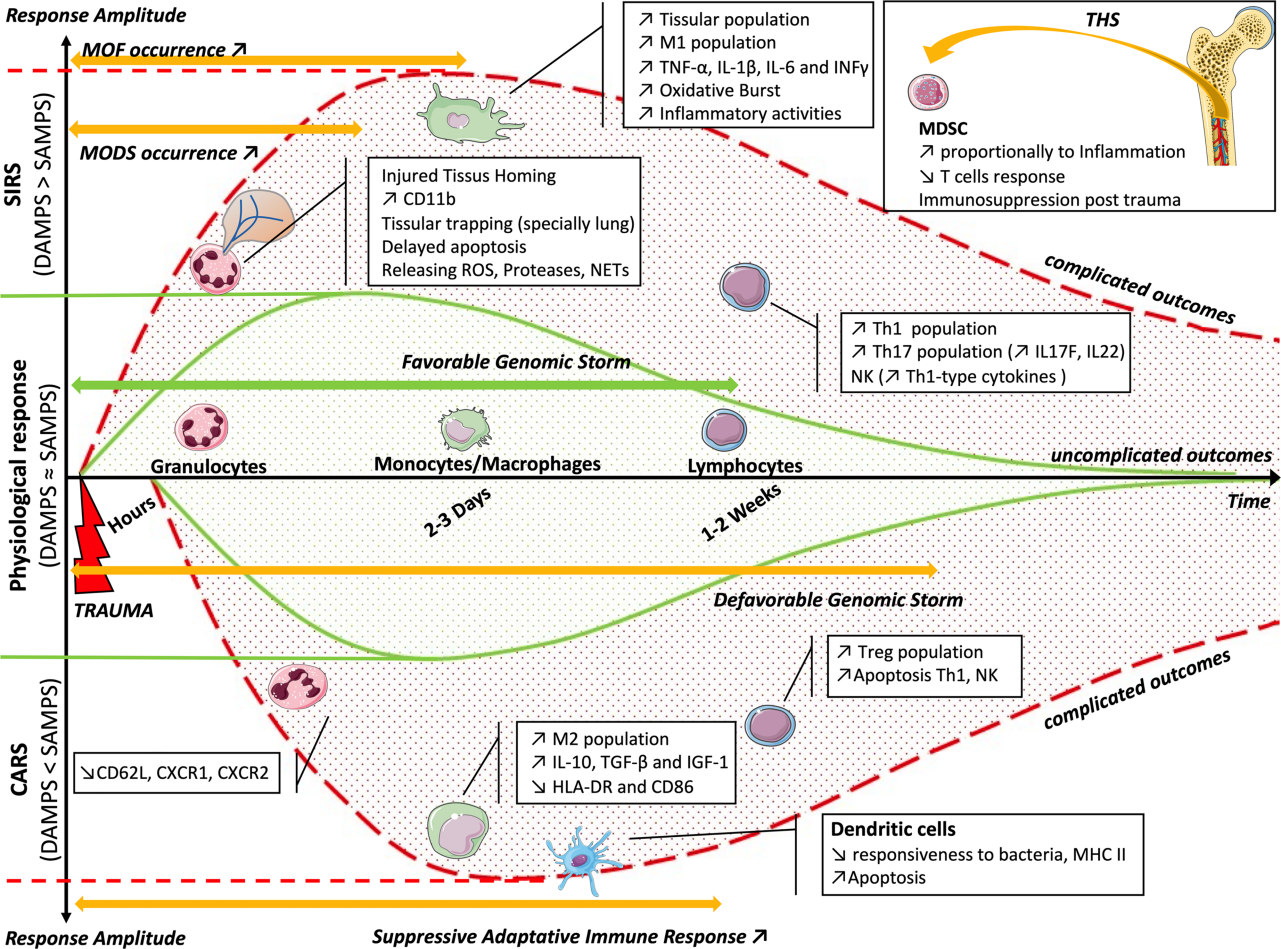
Balance of the inflammatory reaction (CARS or SIRS) as a function of time. The solid green curves represent the physiological response, following the favorable genomic storm and the balance between the effects of DAMPs and SAMPs. In the case of imbalance, the genomic storm becomes unfavorable. The *upper dotted red curve* represents the imbalance toward SIRS, with an increased effect of DAMPs, appearance of MODS and MOF, and cellular modifications. The *lower dotted red curve* represents the imbalance toward CARS, with an increased effect of SAMPs, appearance of suppressive adaptive immune response, and cellular changes. The *box* summarizes bone marrow dysfunction during THS. *CARS*, counterbalancing anti-inflammatory response syndrome; *SIRS*, systemic immune response syndrome; *DAMPs*, damage-associated molecular patterns; *SAMPs*, suppressing inducible DAMPs; *MODS*, multi-organ dysfunction syndrome; *MOF*, multi-organ failure; *THS*, traumatic hemorrhagic shock.

A leukocyte “genomic storm” occurs in critically injured patients, in which up to 80% of the leukocyte transcripts were altered in the first 12 h. It activates a large number of inflammatory mediators or pattern recognition receptors, but also suppresses genes involved in antigen presentation, T-cell proliferation and apoptosis, T-cell receptor function, or NK cell function (**Figure 2**). The unfavorable clinical course of the patients correlates with a higher and longer duration of expression of these genes (28 days, against 7–14 days for a favorable course), but not with the expression pattern (134). These results are consistent with another study describing an increase in blood Th17 CD4^+^ T cells and peripheral monocytes, as well as changes in the NK profile, and the plasma increase in IL-17F and IL-22, TNF-α, IFN-γ, and MCP-1 at 5 days of trauma (135). This suggests that it is illusory to imagine finding a specific marker or a single therapeutic agent that allows avoiding complicated outcomes of patients.

There is a concomitant and synchronous evolution of SIRS and CARS. To restore homeostasis, their evolution must be mirrored. If not, there is an imbalance on the SIRS side and, therefore, the appearance of deregulated inflammation, and even an MOF, or there is an imbalance on the CARS side and the occurrence of infection or delayed healing.

This balance could be the target of therapeutic strategies and help improve the prognosis of patients in the medium and long term after THS (**Figure 2**). Cell therapy or therapy by EVs could therefore be an interesting future strategy in this field.

#### 2.3.3 Microvascular Dysfunction, Endotheliopathy, and Coagulopathy

##### 2.3.3.1 Microvascular Dysfunction

**Figure 3:**
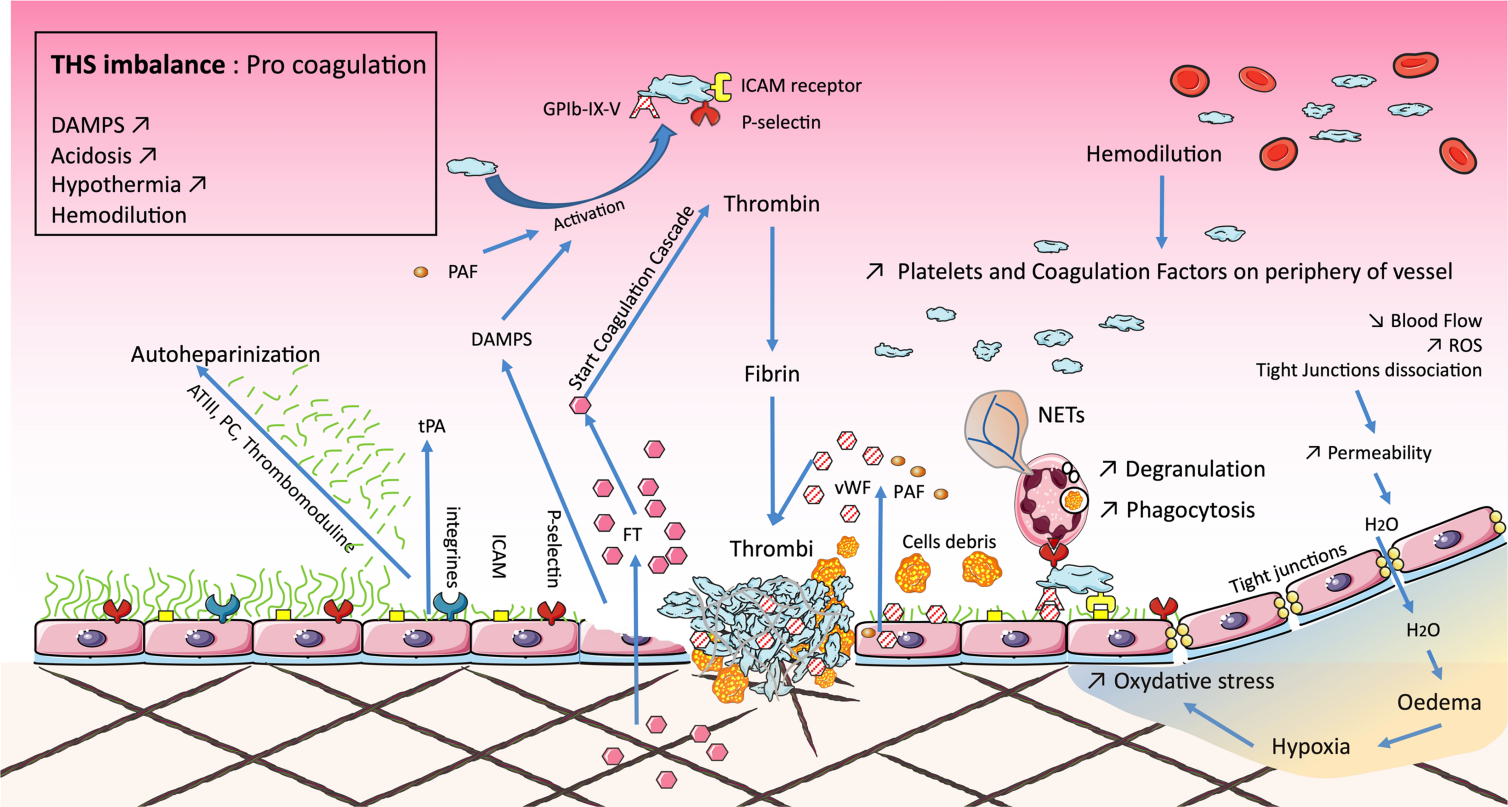
Microvascular dysfunction occurring during traumatic hemorrhagic shock (THS) induces the permeability of tight junctions, responsible for edema, increased oxidative stress, and, ultimately, local inflammation. Endotheliopathy is either direct from tissue damage or secondary to microvascular dysfunction. Endothelial damage degrades the glycocalyx resulting in local autoheparinization. The shedding of the glycocalyx exposes integrins and selectins, promoting the adhesion of platelets and polynuclear neutrophils. Their association stimulates endothelial cells, which release factors such as sCD40L, von Willebrand factor (vWF), and platelet-activating factor (PAF). Endothelial damage is also associated with the release of damage-associated molecular patterns (DAMPs) and tissue factor (TF). This activates the coagulation cascade reducing the downstream blood flow, forming the bed of coagulopathy in 15% of cases, the latter leading to disseminated intravascular coagulopathy (DIC). These phenomena are associated with the lethal triad: coagulopathy, acidosis, and hypothermia.

Microcirculation is made up of three levels: arterioles, capillaries, and venules. All three are affected during THS. The vasoconstriction induced by epinephrine maintains local hypoxia and limits tissue exchange and, therefore, the clearance of lactic acid, for example. This association—coagulopathy, inflammation, anaerobiosis, and oxidation—promotes endotheliopathy (3). In this case, the arteriolar endothelium exhibits a dysfunction in relaxation linked to the local overproduction of ROS by CD11/CD18^+^ cells. In the capillaries, there is an adhesion of activated leukocytes to damaged endothelial cells. There is also a local exudate (**Figure 3**).

The endothelium of post-capillary venules plays a key role in the onset of complications secondary to THS. Firstly, ROS cause complement (C5) activation and the production of several factors (PAF and leukotriene B4), which are able to induce the adhesion and activation of leukocytes on the endothelium. ROS also induce the release of Weibel–Palade bodies, which are large endothelial vesicles that stock von Willebrand factor (vWF) and P-selectin. Then, ROS lead to the production, *via* the NFκB and AP-1 pathways, of E-selectin, intercellular adhesion molecules (ICAM), or even vascular cell adhesion molecules (VCAM). These elements allow the adhesion and diapedesis of CD11/CD18^+^ activated cells such as neutrophils. The inflammatory response is amplified by mast cells and macrophages, which release inflammatory mediators like TNF-α, nitric oxide (NO), histamine, or ROS. All these elements limit downstream blood flow, called microcirculation failure (136, 137).

##### 2.3.3.2 Endotheliopathy

Ischemia and inflammation often result in the disruption of endothelial tight junctions, adherent junctions and glycocalyx components (138–140). The decrease in blood flow is a mechanical stimulus inducing the activation of the adhesion molecule PECAM, vascular endothelial growth factor (VEGF) receptors, and VE-cadherin, which results in the depolarization of the endothelial cell membrane and subsequent ROS generation. These events finally disrupt the integrity of the endothelial cell–cell junction and compromise the endothelial barrier, leading to hyperpermeability (141).

The glycocalyx is an intravascular coat composed of glycosaminoglycans (e.g., heparan sulfate) and proteoglycans (e.g., syndecan) (142). The thickness of the glycocalyx decreases during hemorrhagic shock, in proportion to the reduction in blood flow (143). During I/R, glycocalyx shedding increases the circulating blood concentrations of syndecan-1 (which is highly associated with mortality) (144) and heparan sulfate (145). This results in the exposure of the injured endothelium to pro-inflammatory leukocytes, leading to the alteration of its structural integrity and hyperpermeability (146). Activated neutrophils cause glycocalyx disruption during trauma because they release proteolytic enzymes such as neutrophil elastase and degranulation, which promotes local inducible nitric oxide synthase (iNOS) or ROS synthesis (143).

##### 2.3.3.3 Coagulopathy

Coagulopathy occurs in up to 15% of THS patients. It worsens the bleeding and is associated with excess mortality (139, 147). Tissue factor (TF) is the key element in initiating the coagulation cascade. Tissue damage exposes both TF and collagen, capable of binding factor VII and vWF (148), respectively, and initiating coagulation. At the damaged vascular site, the platelets come into contact with the thrombin formed during the initiation phase of the coagulation cascade and are then massively activated. Activated endothelial cells become procoagulant by secretion of plasminogen activator inhibitor-1 (PAI-1). Moreover, the damaged glycocalyx exposes P-selectin or ICAM-1, favoring platelet and neutrophil adhesion, respectively. In turn, neutrophils promote local fibrin activation and platelet adhesion (143). Observational data suggest a correlation between high levels of circulating syndecan-1 and higher catecholamines, IL-6, IL-10, histone-complexed DNA fragments, HMGB1, thrombomodulin, D-dimer, tissue plasminogen activator (tPA), and urokinase plasminogen activator and a threefold increased mortality (139). In addition, hypotension and hypovolemia during THS cause the release of tPA by endothelial cells (3). This could limit the procoagulant effects of the activated endothelium proteins (e.g., protein C and protein S), which inhibit the coagulation pathways and prevent an inappropriate extension of coagulation beyond the damaged vascular site. Nevertheless, this equilibrium may be broken and trigger trauma-associated coagulopathy. As previously described in the literature (139), there is a continuum between local and initial coagulopathy and disseminated intravascular coagulopathy (DIC), which appears later (hours/day). This DIC is the consequence of extensive trauma, overwhelmed anticoagulant capacity, and major inflammation. Coagulopathy is aggravated as part of the lethal triad *via* acidosis and hypothermia, which are traumatic or iatrogenic, but also by hypovolemia (149–151).

### 2.4 Multiple Organ Failure

MOF is defined as alterations in the function of at least two organ systems, ranging from mild dysfunction to irreversible failure. Risk factors are related to the severity, type, and distribution of injuries (thoracic trauma), as well as the duration of hemorrhagic shock (152). As described previously, the pathogenesis of MOF is complex, with interrelated mechanisms involving neurohumoral and cellular cascades leading to generalized inflammatory reaction, capillary damage and permeability, interstitial edema, and, finally, organ dysfunction/failure (153). MOF should be distinguished from multi-organ dysfunction syndrome (MODS), which occurs frequently during resuscitation. Much of these early organ dysfunctions return to normal within 48 h of injury. The peak of MOF occurs within the first 3 days after injury. Disparate patterns were described: early MOF occurring within the first 3 days post-injury depending on shock severity, carrying high mortality, or late MOF whose incidence increases with age (154, 155). A retrospective study showed that lung failure was the most common organ failure, whereas cardiac and pulmonary system dysfunction decreased and renal and liver failures persisted at similar levels (155). Liver, kidney, or gastrointestinal tract injuries are directly linked to blood flow redistribution to vital organs such as the brain and heart after THS (155, 156).

A large number of scoring systems have been proposed to define MOF, without gold standard. All scoring systems [Denver, Marshall, and Sequential Organ Failure Assessment (SOFA)] include at least the monitoring of cardiac (e.g., mean arterial pressure), respiratory (e.g., PaO_2_/FiO_2_), hepatic (e.g., bilirubin), and renal (e.g., creatinine) functions (157, 158). Serum cytokine expression evaluated each 4 h during 24 h on 48 trauma patients revealed six candidate predictors of MOF occurrence: CXCL10, macrophage inflammatory protein-1 (MIP-1), IL-10, IL-6, IL-1Ra, and eotaxin (159), and the IL-4, IL-6, IL-8, and TNF-α levels are predictors of unfavorable outcomes (160).

However, depending on the type of scoring used and the classification of patients, retrospective studies can have very different conclusions. For example, over the years from around 2000 to 2010, some indicate a decrease in the incidence of MOFs with a MOF-related death rate that did not change. In contrast, others observed a significant increase of MOF prevalence and a decrease of mortality after multiple trauma and notably in the subgroup with MOF (84, 155).

#### 2.4.1 THS-Induced Intestinal Injury

The gastrointestinal tract and the tissues vascularized by the superior mesenteric artery are particularly sensitive to reduced blood perfusion (156). The loss of gut barrier integrity is hypothesized to be the “motor” of MOF by allowing the translocation of organisms from the external environment (including not only bacteria but also proteolytic enzymes) and by limiting systemic access for necessary nutrients (161, 162). Therefore, prevention of gut injury associated with intestinal ischemia could be a key therapeutic strategy. The decrease of mesenteric perfusion after THS leads to hypoxia of the villi (73). DAMPs govern the activation of resident leukocytes, the recruitment of circulating leukocytes, and also the activation of local and systemic complement (45, 82, 163). Inflammatory response, ROS production, and intraluminal pancreatic proteases also lead to mucus layer injury (164–166). Loss of the mucus layer was associated with increased gut permeability (164, 165, 167). Critical illness has a profound effect on the number of cells in the mucosal immune system (161). The lamina propria contains enteric glial cells (EGCs), and DCs; they can both recognize DAMPs and pathogen-associated molecular pattern molecules (PAMPs). EGCs are central in the homeostasis of the intestinal epithelium (168). Moreover, tissue damage can drive the dysregulation of pro-inflammatory group 3 innate lymphoid cell (ILC3s) response, which can contribute to immunopathology (169). In contrast, successful integration of environmental cues by ILC3s allows homeostasis of the gut–blood barrier by the production of IL-22. This route allows the restoration of local intestinal homeostasis after trauma (73, 169). It was demonstrated that severe THS caused an increase in bacterial translocation from the gut to the blood and organs, such as the liver and spleen. Moreover, it induces a modification toward a naive Th2 phenotype of CD4^+^ and a tolerogenic phenotype of DC in mesenteric lymphatic nodes, which is consistent with the clinical forms of immunosuppression observed in severe patients (170). Interestingly, a recent study of gut I/R showed that mice displayed a significant inflammatory response with neutrophil infiltration into mucosal areas, but also in the lung. Mesenteric lymph duct ligation, which had no effect on gut injury, attenuated lung injury following gut I/R. This study highlights the central role of the gut in the development of systemic inflammation and MOF, including acute lung injury (ALI) (171). Thus, the digestive tract can be both an instigator and a victim of MOF (172).

#### 2.4.2 THS-Induced ALI

ALI and acute respiratory distress syndrome (ARDS) are serious complications of traumatic injury. ALI/ARDS constitute a pathophysiological continuum that is defined by a lung disease with acute onset, non-cardiac, diffuse bilateral pulmonary infiltrates and a PaO_2_/FiO_2_ ≤300 for ALI or ≤200 for ARDS (173).

In a recent study, 30% of patients developed ARDS as a result of trauma, with a death rate three times higher. Lung damage can be caused by pulmonary contusion, shock, administration of blood products including platelets, and an element of volume overload that can occur in the presence of increased pulmonary vascular permeability (174). Following hemorrhagic shock, neutrophils (activated *via* NF-kB and NLRP3 signaling) and macrophages (*via* HMGB1/TLR4 pathway) induce pulmonary inflammation (175). Moreover, in a model of THS lymph-induced ALI, the lung injury was totally abrogated in neutrophil-depleted animals (176). This inflammation locally damaged tight junctions and endothelial cells and ultimately led to the production of edema and the deterioration of capillary alveolar exchanges (107). Furthermore, pulmonary edema is aggravated by the decreased production of surfactant by injured endothelial cells (177).

#### 2.4.3 THS-Induced Acute Liver Injury

During hemorrhage, the spongy hepatic structure and vascular response, modulated by hepatic sympathetic nerves, could temporarily compensate for the volume of blood lost. Moreover, hepatic glycogenosis could also compensate for hypovolemia by an osmotic effect toward the vessels (178). Nevertheless, THS inducing liver ischemia rapidly leads to endothelial and hepatocyte cell death (179). The diagnostic evaluation includes a combination of biochemical tests with, for example, the determination of serum hepatobiliary enzymatic activities [alanine aminotransferase (ALT) and aspartate aminotransferase (AST)] and gamma-glutamyl transpeptidase (GGT). Histopathologic changes include cellular swelling, vacuolization, endothelial cell disruption, neutrophil infiltration, and hepatocellular necrosis (180, 181). Following THS, the number of activated Ito cells (perisinusoidal fat-storing cells, stellate cells, and lipocytes) and Kupffer cells (KCs, resident hepatic macrophages) are increased. Activated KCs migrate from hepatic sinusoids into the injury areas, increase phagocytosis, and release ROS and various cytokines such as TNF-α, IL-1, IL-6, or IFN-γ (34, 181). This results notably in neutrophil activation and their sequestration in different vascular beds of the liver (156, 180). Neutrophil release NETs, proteases, and ROS, inducing hepatocyte injury and their release of DAMPs (133, 182). DAMPs (the most described in the liver is HMGB1) and also the complement pathway can activate KCs. The pro-inflammatory cytokines and ROS released by activated KCs also exert cytotoxic effects by inducing changes in the cell membrane receptors of hepatocytes and endothelial cells. They also activate other KCs and produce chemotactic factors for neutrophils and CD4^+^ lymphocytes (181, 183–187), which aggravate microvascular/hepatocellular injury by the formation of cellular thrombi (133).

#### 2.4.4 THS-Induced AKI

The incidence of acute kidney injury (AKI) is indicated at 13% in trauma and increases to 42.5% in THS; 96% of AKI appear within the first 5 days (188). AKI is the clinical endpoint of multiple processes and results in a decrease in the GFR, which is a measure of global renal function. The injury mechanisms identified are I/R, inflammation, and rhabdomyolysis. In the nephron, the glomerulus is exposed to vasoconstriction of the afferent glomerulus artery, resulting in a decrease in the GFR by injury of the glomerular–blood barrier. Cellular debris can precipitate in the tubule, further decreasing renal filtration and reabsorption. I/R injury is among the most common causes of AKI, and the underlying pathogenesis involves injury to the nephron by both ischemia and oxidative stress survival/death mechanisms. Proximal renal tubular cells along the nephron segments are particularly sensitive to hypoxia. One of the early events in renal I/R is the activation of the endothelium (increased expressions of E-selectin, ICAM-1, and CX3CL1), increasing vascular permeability and promoting leukocyte extravasation. Moreover, tubular epithelial cells increase complement binding and upregulate TLRs, leading to cytokine/chemokine production. A study in patients suffering from AKI post-blunt trauma showed a rapid increase in concentrations on D0 (time of measurements after injury within the first 12 h = D0, 24–96 h = D1–D4, and ≥96 h ≥ D4) in inflammatory factors [e.g., IL-8, MCP-1 (alias CCL2), and IL-6] and anti-inflammatory factor (e.g., IL-1ra), followed by a drop on D4, in IL-1ra, IL-4, and IL-6 (189). In the tubules, neutrophils are observed between 3 and 24 h, followed by an ascending plateau up to 72 h after I/R injury (190). Macrophages are recruited at D1, with a peak at D5. The M1 is the dominant population from D1 to D3, then the M2 from D5 to D7. The authors demonstrated the role of M1 in the onset of tissue lesions and more of M2 in tubular repair (191). The I/R model also induces a maturation of the DC phenotypes and their production of TNF-α, IL-6, or MCP-1 in the first 24 h (192). Moreover, the injured epithelium releases fraktaline, which recruits more DCs (193). Finally, the kidney disposes type 2 innate lymphoid cells (ILC-2 cells), which appear to be involved in the anti-inflammatory phase. ILC-2 releases IL-4 and IL-13, allowing the polarization of macrophages and lymphocytes to M2 and Th2/Treg phenotypes, respectively (194). Finally, rhabdomyolysis is a classic complication of severe trauma ranging from the elevation of serum myoglobin and creatinine kinase (CK) activity to AKI and disseminated intravascular coagulation. It induces disturbances in intracellular ionic gradients, leading to increased concentrations of intracellular Ca^2+^. The pathogenesis of AKI by rhabdomyolysis involves myoglobin-induced intrarenal vasoconstriction, direct ischemic injury, and tubular obstruction (195). Moreover, in a model of rhabdomyolysis-induced AKI, the heme-activated platelets enhanced the production of macrophage extracellular traps (METs) by increasing intracellular ROS generation and histone citrullination (196). There is a need today to find new therapies to prevent/treat kidney damage in order to avoid the clinical consequences associated with AKI and progress to chronic renal failure (197).

## 3 Current Management of THS

### 3.1 Current Support

The current management of hemorrhagic shock is based on two main pillars: stopping the bleeding and damage control resuscitation. This is applied during the pre-hospital and intrahospital phases (198). Bleeding control is first achieved by local compression, placing tourniquets or hemostatic dressings. The definitive management of these wounds requires surgical hemostasis (150, 198). The aim of damage control resuscitation is to maintain permissive hypotension (80–90 mmHg) as long as surgical hemostasis is not achieved; it is a compromise between tissue perfusion and aggressive resuscitation with high doses of fluids (199). Moreover, this limits hemodilution by overfilling, which helps maintain DO_2_ above the critical limit (<8/10 ml min^−1^ kg^−1^) (28, 198).

Preserving blood pressure begins with vascular filling. It is recommended to use a plasma first (200). Treatment with plasma during massive bleeding allows restoration of the glycocalyx (145). The use of vasopressors or sympathomimetics is only recommended as a second-line treatment (198, 201–203). Hemoglobinemia is not the only criterion for optimal transfusion. It is recommended to start the plasma transfusion at the same time, with a plasma/blood cell ratio between 1:2 and 1:1. Platelet infusion should be administered to maintain a minimum count, depending on the clinical situation (150, 198). To finish, tranexamic acid must be used before the third hour after THS for anti-fibrinolytic action. Other treatments such as coagulation factor concentrate, fibrinogen supplementation, or calcium supplementation could be used against coagulopathy (150, 198).

### 3.2 Frontiers in Current Management

The complexity and heterogeneity of the multiple factors involved in the pathophysiology of THS can give rise to MOF despite constant improvement in patient care. Deregulations of the immune system are at the heart of systemic deregulations after injury; therefore, modulating the immune response is a promising therapeutic strategy for preventing the complications of THS.

Preclinical and clinical proof-of-concept studies have analyzed the efficacy of new and emerging therapeutic candidates in the context of individual organ failure. Although informative, these studies do not address the full complexity of THS. Hence, hypothesis-driven research studies targeting the multi-organ dysfunction of THS are urgently needed. The therapeutic potential of MSC therapy has been well characterized and demonstrated to improve tissue function and regeneration. The established immunomodulation capacity and ability to restore tissue damage may also be applied in the treatment of THS-induced MOF. THS is a life-threatening emergency requiring immediate medical intervention. While cell-based therapy carries multiple advantages, the drawback is the delay of the supply of MSCs that require *in vitro* expansion and the complex storage and transport before administration. EVs, on the other hand, are secretory products of MCS. The major advantage of using cell derivatives rather than cells is the immediate availability of the product, which may be prepared, amplified, characterized, and easily stored for future use in the emergency context of THS patients. Existing evidence indicates that MSC-derived EVs are able to prevent immunological disturbances that lead to organ failure.

## 4 MSC-Derived Extracellular Vesicles: Toward Cell-Free Therapeutic Applications

### 4.1 Mesenchymal Stromal Cells

MSCs have been described since 1970 (204). These cells of mesenchymal origin have been found in both perinatal tissues and numerous adult tissues (205). Although isolated from various tissues, they share common properties described in 2006 by the International Society for Cellular Therapy (ISCT), which proposed minimal criteria to define MSCs. These plastic-adherent fibroblastic-like cells express a panel of antigenic surface markers (positive for CD73, CD90, and CD105 and negative for hematopoietic markers) and have an *in vitro* multipotency capacity in the three canonical pathways: osteoblastic, chondroblastic, and adipocytic (206). They have many capacities: trophic support and immunomodulation, as described above, but also anti-apoptosis, pro-angiogenesis, or even antioxidation (207). MSCs were first described as key regulators of the HSC niche homeostasis. Later, in the 2000s, it has been described that allogeneic MSC transplantation given intravenously is well tolerated (11), can promote hematopoietic engraftment (208), accelerate lymphocyte recovery (209), reduce the risk of graft failure, and reduce the incidence of GvHD (12, 210). MSCs can modulate innate immunity by promoting the repolarization of monocytes and macrophages from a type 1 (pro-inflammatory) to a type 2 (anti-inflammatory) (211), by suppressing the proliferation, cytokine secretion, and NK cell cytotoxicity (212), and by inhibiting the maturation and migration of DCs (213), as well as modulate polymorphonuclear cell apoptosis and activity (214). MSCs can also modulate both adaptive immune effector activity by inhibition of T-cell (215) and B-cell (216) functions. These data open the way to their utilization as cell therapy products in degenerative and/or inflammatory diseases lacking appropriate treatments (217). Presently, hundreds of clinical trials are using MSCs to evaluate their therapeutic effects in numerous severe diseases (217). The first clinical trial in this context, using the systemic administration of allogeneic MSCs, did not exacerbate the elevated cytokine levels in the plasma of septic shock patients, consistent with a safe response. This cohort also revealed patient-specific and dose-dependent perturbations in cytokines, including an early but transient dampening of pro-inflammatory cytokines (218).

This immunomodulation potential has been extensively documented. Caplan and Dennis (219), in 2006, postulated that MSCs could mediate their therapeutic activity *via* the secretion of soluble factors such as prostaglandin E2 (40), IL-1 receptor antagonist (IL-1RA) (220), TGF-β (221), hepatocyte growth factor (HGF) (222), indoleamine 2,3-dioxygenase (IDO) (223), or tumor necrosis factor-stimulated gene 6 (TSG-6) (224–226) rather than by direct cellular interactions. In 2007, it was then demonstrated that MSC-conditioned media rich in small EVs could exert cardioprotective effects in a myocardial infarction model (227). Another team described the beneficial effects of MSC-conditioned media enriched with larger EVs in a mouse model of AKI (228). Consequently, today, there is a growing interest in MSC-derived EVs (MSC-EVs). More recently, it was also demonstrated that MSC-EVs can be a promising therapy for preventing chronic GvHD by exhibiting potent immunomodulatory effects (229, 230). Moreover, in several preclinical studies, it was shown that MSC-EV therapy reduced inflammation in kidney injury animal models (231) and decreased the inflammatory cell influx, altering alveolar macrophages toward an anti-inflammatory phenotype in lung injury models (232, 233) or the pro-inflammatory cytokine messenger RNA (mRNA) levels in liver injury (234). Finally, it is important to understand that MSCs are sensitive to their environment. Their properties and those of their by-products may vary depending on growing conditions, known as the concept of “priming” (235, 236). Stimulating MSCs with pro-inflammatory cytokines such as IFN-γ, TNF-α, IL-1α, or IL-1β induces the secretion of soluble or EV-encapsulated anti-inflammatory factors (237–240). Interestingly, the secretome of MSCs primed with IL-1β and the sera of polytrauma patients share important characteristics. IL-1β priming enhances the secretion of pro-inflammatory and pro-angiogenic factors (IL-6 and VEGFA) and chemokines (CXCL1 and CCL2). Moreover, MSC-IL-1β priming may improve their therapeutic effects by inducting cell adhesion molecules and anti-inflammatory and anti-fibrotic molecules (241).

The few studies using MSCs in THS showed that their administration early after hemorrhagic +/− traumatic shock limited vascular permeability by preserving the barrier junction proteins (VE-cadherin, claudin-1, and occludin-1), inhibiting the expressions of leukocyte adhesion molecules (VCAM-1 and ICAM-1) on endothelial cells, and decreasing both serum concentrations of inflammatory molecules and CD68- and MPO-positive cell tissue infiltration (17). We recently showed that IL-1β-primed MSCs attenuated hemorrhagic shock-induced early hepatic and kidney injury and dysfunction and reduced the SIRS/CARS syndrome, as shown by the decreases in the plasma cytokine concentrations and the phenotypic activation of circulating CD11bc^+^ cells (242). MSCs would also prevent the decrease in hematopoietic progenitors induced by THS in the bone marrow (15, 17, 243). Whether the use of MSCs could alleviate or potentially exacerbate THS-induced coagulopathy is unclear. MSCs express TF (244, 245) and phosphatidylserine (246), which are thrombogenic and tend to increase the clotting rates. MSCs can therefore behave as beneficial hemostatic agents, but can also be excessively procoagulant, depending on the dose, the time of administration, and the method of preparation, which, in this case, may require the concomitant administration of anticoagulants to prevent venous thromboembolism or disseminated coagulopathy (247). Moreover, prothrombotic factors on their surface could trigger the instant blood-mediated inflammatory reaction (IBMIR) after blood exposure. IBMIR is characterized by the activation of complement/coagulation cascades, the binding of activated platelets to the MSCs, and clot infiltration by neutrophils and monocytes, which could lead to cell destruction. It is important to note that the induction of IBMIR depends on the MSC source and the dose administered and increases when their *in vitro* expansion has been high (passages 5 to 8) (245). This means that the choice of the modes of preparation and administration of MSCs can modulate their thrombotic activity. In contrast, cultured fibrin-embedded human MSCs can dissolve the surrounding fibrin mesh. This fibrinolytic capacity may be related to the transcriptional expression of the urokinase plasminogen activator (uPA) and its receptor (uPAR), the tPA, and the PAI (248). In conclusion, MSCs are being employed as an experimental therapy in a variety of human diseases and represent an important hope in the context of lesions induced by THS. They act on several biological processes including inflammation and reprogramming of immune cells, but also by the activation of endogenous repair pathways. Current dogma indicates that they improve disease through the secretion of paracrine-acting factors and, more recently, *via* the production of EVs.

In the emergency context of THS, requiring very quick availability of the therapeutic product, a ready-to-use EV solution, appears to be a particularly interesting innovative strategy.

### 4.2 MSC-Derived EVs

#### 4.2.1 EV Definition

Cells use multiple and sophisticated modes of communication. Besides direct cellular communication through the expression of cell surface markers, they communicate not only by the secretion of soluble molecules but also *via* the production of EVs. The term “extracellular vesicle” corresponds to a generic term that refers to particles naturally released from the cells, delineated by a lipid bilayer, and are devoid of replicative activity (i.e., without functional nucleus). The three main EV subtypes found in the literature include microvesicles (MVs; also known as microparticles or ectosomes), exosomes (exo), and apoptotic bodies. They are characterized by their size (small vesicles, <100–200 nm; medium/large, >200 nm), density, cellular origin, and their biochemical composition (tetraspanin, annexin V, etc.) or according to their biogenesis process (249). The biogenesis of small vesicles (exosomes) occurs in early endosomes; then, during the process of maturation, the early endosomes become endosomes or multivesicular bodies and accumulate intraluminal vesicles, which can either be degraded by lysosomes or released as exosomes in the extracellular space. The biogenesis of medium/large vesicles (MVs) occurs *via* the direct budding of the cell membrane and are released into the extracellular space (250). Apoptotic bodies are large-sized vesicles that specifically originate from apoptotic cells (251). These EVs contain bioactive soluble molecules (mRNA, miRNA, proteins, lipids, etc.) and membrane-bound molecules (CDs and enzymes). The most currently available EV isolation methods [ultracentrifugation, tangential filtration, immunocapture, or precipitation (252), including those used for clinical grade isolation] do not allow the precise isolation or purification of a specific EV subpopulation (exo or MVs) (253–255). Therefore, the International Society for Extracellular Vesicles (ISEV) has suggested minimal information for studies of extracellular vesicles (MISEV). These guidelines indicate that “EV” remains a collective term describing a complex continuum of vesicles of different sizes and composition and resulting from various mechanisms of formation and release (249). Moreover, in most cases, EV preparations are composed of different vesicles and a greater or a lesser amount of soluble proteins that may participate in the biological activity of the final product. We must therefore also take into consideration the heterogeneity of the final preparations used in the different studies, which mostly include soluble factors. The most suitable term would ultimately be “EV-enriched secretome” rather than “EVs.”

Intercellular communication *via* extracellular cargo is highly conserved across species (from bacteria to human); therefore, EVs are likely to be a highly efficient, robust, and economic manner of exchanging information between cells (256).

The specific combinations of molecules in EVs generally reflect the unique characteristics of their original cells and influence their functional properties; therefore, these EVs could recapitulate most effects of the cells from which they originate from and be used as substitutes of those cells in therapeutic objectives (18). EVs can be harvested from all body fluids and take part in many physiological and pathophysiological processes (18). Indeed, EVs are frequently produced in greater abundance in stressed than in unstressed cells; therefore, they can promote the activation of immune cells such as macrophages, which can, in turn, also release EVs and soluble factors and promote stress cell and tissue inflammation and injury (257).

The most extensive studies on EV-mediated communication have been performed between tumor and immune cells and between different types of immune cells. Currently, dendritic cells and mesenchymal stromal cells are the sources for which the prospects for clinical use in humans are most advanced. Since the first descriptions of the therapeutic potential of MSC-EVs in AKI and MI models (227, 228), many studies have addressed the therapeutic functions of MSC-EVs. They could provide new therapeutics and have to be better described and understood (249, 258).

#### 4.2.2 Immunomodulatory Capacity of MSC-EVs

##### 4.2.2.1 Interaction Between MSC-EVs and Innate Immune Cells

As described above, MSCs release a unique signature of proteins (259), lipids (260), and membrane receptors or various types of nucleic acids through EVs (258), which participate in the protection and the regeneration process of damaged cells notably by mitigating the immune response (261, 262). Proteome analysis of MSC-EVs provided by the ExoCarta database showed that the MSC-EV proteome is rich in IL-10, HGF, and leukemia inhibitory factor (LIF) anti-inflammatory cytokines. Moreover, some cytokines, chemokines, and chemokine receptors involved in immune cell recruitment, cell migration, immunosuppression, or neutrophil degranulation, such as CCL2, VEGFC, CCL20, as well as chemokine ligand 2 (CXCL2), CXCL8, CXCL16, defensin α1, HERC5, and IFITM2, are also expressed (261). Similarly, they carry microRNAs (miRNAs) involved in immune function, like miR-146b, identified as an IL-10 effector on macrophages by targeting the TLR4 pathway (263), or miR-181c, which also decreases the expression of TLR4 and the activation of the NF-κB pathway (264). In addition, the pro-inflammatory priming of MSCs, for example by TNF-α and IFN-γ, generates modifications of the protein content and the transcripts of MSC-EVs, notably a greater expression of COX2 leading to an increased release of PGE2, which could promote their anti-inflammatory activity (260). Hypoxia also modulates the MSC-EV miRNA expression profile with notably significant overexpressions of miR-223 and miR-146b, which are implicated in the inflammatory phase of the healing process (265).

Concerning the anti-inflammatory effects on DCs, the authors described 49 miRNAs enriched in MSC-EVs, including miR-21-5p, miR-142-3p, miR-223-3p, and miR-126-3p, known for their role in DC maturation and function (266).

Macrophages have an important role in the inflammatory phase firstly by their pro-inflammatory phenotype and then by their switch to a pro-resolving, anti-inflammatory phenotype. A study in which unfractionated PBMCs were co-cultured with PKH26^+^-MSC-derived EVs showed that EVs were mostly internalized by monocytes and scarcely by lymphocytes after 24 h or 4 days, but inflammatory priming of MSCs increases EV internalization by lymphocytes (267). It was already described that PBMC or macrophage co-culture with adipose-derived MSC-Exo (MSC-derived exosomes) could induce M2 macrophage polarization (265, 268). MSC-EVs also inhibited TNF-α and IL-6 production by inflammatory glial cells and limited their activation (loss of CD45 and CD11b expressions) and induction of CCL2, one of the membrane markers of M2 polarization (269). Finally, bone marrow MSC-EVs could also downregulate the production of IL-23 and IL-22 by macrophages and pro-inflammatory cytokines, inducing Th17 effector T cells. Therefore, MSC-EV-educated macrophages could promote resolution *via* the decrease of Th17 pathogenicity (270).

##### 4.2.2.2 Interaction Between MSC-EVs and Adaptive Immune Cells

MSC-EVs limit the proliferation and differentiation of activated CD4^+^ and CD8^+^ lymphocytes (271). They induce CD3^+^ and CD4^+^ lymphocyte apoptosis and increase the Treg/T effector balance (272) by promoting the passage from Th1/Th17 to Th2 (273–275). Otherwise, in co-culture with activated PBMCs, MSC-EVs inhibit the secretion of TNF-α and IL-1β, but increase the concentrations of TGF-β (276) and IL-10 in the co-culture medium (272). As described above, monocytes and, to a lesser extent, lymphocytes were able to internalize PKH26^+^-MSC-derived EVs. Interestingly, the uptake of MSC-derived EVs occurred in resting but mostly in activated immune effector cells, allowing presumption of a possible role of EVs in immunosuppression, and the inhibition of EV secretion impairs the immunosuppressive capacities of MSCs. Moreover, EV uptake by stimulated B lymphocytes and NK cells is more important than that by T lymphocytes and correlates with the immunosuppressive activity of EV, observed only for B lymphocytes and NK cells, but not for T lymphocytes. Finally, pro-inflammatory priming of MSCs induced an increase in the levels of the anti-inflammatory miRNA-155 and miRNA-146 in both MSCs and their EVs (267, 277, 278). Another study also reported a concentration-dependent immunosuppressive effect of MSC-derived exosomes on B lymphocytes (263).

All these elements show MSC-EVs representing a promising therapy for inflammatory diseases.

## 5 Immuno-Properties of MSC-EVs and MOF

There is significant expanded literature concerning the use of MSC-EVs in multiple preclinical models, in particular on isolated organ damage (**Table 1**). However, no data are currently available on their use in the context of THS. In the following paragraphs, the beneficial effects of MSC-EVs on immunological deregulations and the endothelial dysfunctions of several critical organs injured in MOF are exposed. Otherwise, although inflammation and coagulation are interdependent processes that can initiate a vicious cycle in which each process intensifies the other, the potential benefit of MSC-EVs on coagulopathy has been poorly explored. However, as discussed previously in the section on MSCs, EVs express phosphatidylserine and TF on their surfaces, which were functionally thrombogenic and tended to increase the clotting rates (246) or IBMIR.

**Table 1:** Overview of the applications of mesenchymal stromal cell-derived extracellular vesicles (MSC-EVs) in preclinical experimental studies.

Organ	Model	Microparticles			Priming CSM	Model	Administration			Results	References
		Origins	Type	Purification			Timing	Route	Dose		
GUT	TNBS induced colitis	Human bone marrow derived MSC	EV	ultracentrifugation	/	male Sprague-Dawley	3 days after colon lesions	systemic	50, 100 , or 200µg EV diluted in 1mL	EV ↘ histological lesions, inflammation (expression of TNFα,IL-1β, NF-κBp65, iNOS or COX2↘, and expression of IL-10 ↗), and apoptosis (Cleavage od caspase 3, 8 and 9), ↗antioxydant effect. Dose response effects	Yang et al. (279). doi: 10.1371/journal.pone.0140551
	DSS induced colitis	Dog Adypocytes tissu derived MSC	EV	ultracentrifugation	transfection (TSG-6 siRNA)	C57BL/6 male mice	during the intoxication week at day 1, 3 and 5	intraperitoneal	100 μg of EV diluted en 100μL/mouse	EV ↘ histological lesions, inflammation (expression of TNF-α, IFN-γ, IL-1β or IL-6↘, and expression of IL-10 ↗). Polarization M2 via TSG-6 pathway	An et al. (269). https://doi.org/10.1371/journal.pone.0220756
						vitro : dogs PBMC, LPS stimulated	coculture with EV	/	100 μg/well		
	DSS induced colitis	Dog Adypocytes tissu derived MSC	EV	ultracentrifugation	24 h with TNF-α and IFN-γ	C57BL/6 J male mice	during the intoxication week at day 1, 3 and 5	intraperitoneal	100 μg of EV diluted in 100μL/mouse	Pro inflammatory primed EV, over express immunosuppressive protein (HGF, TSG-6, PGE2or TGF-β). EVs ↘ histological lesions, inflammation, ↗ Tregs, and M2 polarization	An et al. (270) https://doi.org/10.1038/s41598–020–58909–4
						vitro 1: RAW 264.7 - vitro 2: DH82, LPS stimulated - Vitro 3: canine PBMC concavalin A stimutated	coculture with EV	/	50 μg/well		
	DSS induced colitis	Mouse bone marrow derived MSC	EV	ultracentrifugation	/	BALB/c male mice	1 per day, during seven intoxications days	intraperitoneal	50 μg of EVs/mouse	Ev ↘ symptoms, histological lesions, and VEGF-A, IFN-γ, IL-12, TNF-α, CCL-24, or CCL-17 levels. EV ↗ IL-10 and TGF-β levels. EV allow polarization M2.	Cao et al. (280) https://doi.org/10.1016/j.intimp.2019.04.020
						Vitro: macrophage stimulated LPS	coculture with EV	/	100 μg/mL EVs for 24 h		
	DSS induced colitis	Mouse Adypocytes tissu derived MSC	Exosome	exosome isolation kit		C57BL/6 female mice	during the intoxication week at day 2, 4 and 6	intraperitoneal	100 μg exosome diluted in 200 μl	Ev ↘ symptoms, histological lesions, inflammatory cells penetration. In spleen end lymph nodes, Treg, TGF-β, IL-4, and IL-10 ↗and IFN-γ, TNFα, IL-12, or IL-17↘	Heidari et al. (281), https://doi.org/10.1002/jcp.30275
	DSS induced colitis	hUC-MSCs	Exosome	ultracentrifugation	/	male KM mice	during the intoxication the 11 days at 3, 6, and 9	intraperitoneal	400 μg exosomes/mouse	hUC-MSCs-Ev ↘ symptoms, histological lesions, inflammatory cytokines levels (TNF-α, IL-1β, iNOS, IL-6 or IL-7), ↘ macrophages infiltration, and ↗ IL-10 levels, in colon and spleen.	Mao et al. (282) https://doi.org/10.1155/2017/5356760
						vitro : Mice macrophage	coculture with EV	/	exosomes 160 μg/ml		
	TNBS induced colitis	Rat bone marrow derived MSC	EV	ultracentrifugation	/	male Sprague-Dawley	3 days after colon lesions	systemic	50μg /100μg / 200μg EV	Ev ↘ symptoms, histological lesions. Ev limit Th17 polarization via increase H3K27me3 levels. Dose response effects	Chen et al. (283) https://doi.org/10.1016/j.molimm.2019.12.019
	Small bowell transplantation rejection	Rat bone marrow derived MSC	Exosome	Exosome separation kits	transfection (Heme Oxygen-1)	Allograft ( Lewis rat (donnor) Brown Norway rat (Recipient))	/	/	/	HO1-MSC derived exosomes ↘ inflammatory injury , via miR-200b which ↘ Hmgb3 gene expression in intestinal epithelial cells.	Sun et al. (284), https://doi.org/10.1038/s41419-020-2685-8
						vitro : Rat intestinal epithelial cells, inflammation injured wirh TNF-α (100 ng/mL) and lymphocytes	coculture with CSM	/	100 μg/mL Exosome		
	TNBS induced colitis	Rat bone marrow derived MSC	EV	ultracentrifugation	transfection (miR-146a)	male Sprague-Dawley	3 days after colon lesions	systemic	100 μg EV diluted in 1ml	Ev ↘ histological lesions. MiR-146a negatively regulates TRAF6 and IRAK1 and decrease inflammatory (↘TNF-α, IL-6 or IL-1β) via suppressing NF-κB activation pathway.	Wu et al. (285) https://doi.org/10.1016/j.intimp.2018.12.043
	DSS induced colitis	hUC-MSCs	Exosome	ultrafiltration	/	C57BL/6 male mice	during the intoxication at days 3, 6, and 9	systemic	1 mg Exosome	Exosomes ↘ histological lesions, pro-inflammatory factors (IL-1β, IL-6), and ↗ IL-10. miR-326 overexpressed in hucMSC-Ex inhibit neddylation process and NF-κB pathway.	Wang et al. (277) https://doi.org/10.1002/ctm2.113
						vitro : Human colorectal cells LPS stimulated	coculture with exosomes	/	200 μg Exosome		
	DSS induced colitis	Rat bone marrow derived MSC	EV	ultracentrifugation	transfection (EphB2)	male Sprague-Dawley	after the intoxication week at day 8 and 11	systemic	100 μg of EV diluted in 100μL	EphB2-EV ↘ symptoms, histological lesions, inflammation (NF-κB level, TNF-α, IFN-γ, IL-1β, and IL-2 ↘), STAT3 expression, and oxydative stress. EphB2-EV ↗ Treg polarization.	Yu et al. (278) https://doi.org/10.1186/s13287–021–02232–w
	DSS / TNBS induced colitis	hUC-MSCs	Exosome	ExoQuick-TC	transfection (siTSG-6)	C57BL/6 male mice	TNBS: 24h after colon lesions	intraperitoneal	200µg Exosome/mouse	↘ mortality, symptoms, histological lesions, pro-inflammatory cytokines, ↗ anti-inflammatory cytokines, switch toward Th2. Effects via TSG-6	Yang et al. (286), https://doi.org/10.1186/s13287-021-02404-8
							DSS: 5 days after intoxication	intraperitoneal	200µg Exosome/mouse		
LUNG	Traumatic ALI	Rat bone marrow derived MSC	Exosome	exosome EVtant/centrifugation	overexpressed plasmid vectors (miR-124-3p)	male Sprague-Dawley	30 min before procedure	sytemic	25 μg of exosomes	↘ oxidative stress injury, inflammatory response. Mediated by miR-124-3p	Li et al. (287), doi:10.1152/ajplung.00391.2018
	I/R induced ALI	Rat bone marrow derived MSC	Exosome	ultracentrifugation	/	male Sprague-Dawley	end of procedure	sytemic	5 - 10 μg of exosomes	↘ TNF-α, IL-6 and IL-1β . ↘ TLR4 and NF-κB levels in rat lung tissue	Liu et al. (288), doi: 10.7150/ijms.35369
	Histone induced ALI	Mice Adypocytes tissu derived MSC	Exosome	exosome precipitation kit	GW4869 (N-Smase inhibitor)	male C57BL/6 N mice	ADCS Injection 30 min prior to injury	sytemic	3 × 10'5 cells/mice	Exosomes ↘endothelial damage via the PI3K/Akt pathway, modulate by miR-126.	Mizuta et al. (289) https://doi.org/10.1186/s13287–020–02015–9
					/	human umbilical vein endothelial cells exposed to histones	coculture with exosomes	/	/		
	HS induced lung vascular permeability	Human bone marrow derived MSC	EV	ultracentrifugation	/	C57BL/6 male mice	end of HS	systemic	30 μg of EVs	In vivo: ↘ vascular permeability, via cytoskelatal proteins phosphorylation. In vitro, MSC CM but not MSC-EVs prevented thrombin-induced endothelial cell permeability.	Potter et al. (290), doi:10.1097/TA.0000000000001744
						vitro: Human lung microvascular EC cells	coculture with EV	/	/		
	I/R and ex vivo lung perfusion induced lung injury	hUC-MSCs	EV	ultracentrifugation	/	C57BL/6 wild-type mice	1 h before ischemia	intratracheal	MSCs or EVs (1 × 10'6)	↘ edema,neutrophil diapedesis and proinflammatory cytokine (IL-17, TNF-α, HMGB1, CXCL1, MCP-1, IL-6, MIP-1α, ). Immunomodulatory effect.	Stone et al. (291), doi 10.1186/s12931-017-0704-9
						vitro 1: murine iNKT cells and macrophages - vitro 2: mice primary lung microvascular endothelial cells	coculture with EV	/	/		
LIVER	I/R induced liver injury	Mouse bone marrow derived MSC	EV	ultracentrifugation	/	C57BL/6 mice	30 min before ischemia	systemic	2 × 10'10 EV diluted en 200µL	Ev ↘ histological lesions, apoptosis, hepatic enzymes releasing (AST, ALT, BUN), NFκB and ROS activity. Immunomodulatory effect (TNF-α, IL-1α, IL-1β, IL-6, IL-12, or IFNγ↘, and CXCL1 or MCP-1 ↗).	Haga et al. (292), doi: 10.1002/lt.24770
						vitro: AML12 and hypoxia culture	coculture with EV	/	1,8 ×10'8 EV		
	I/R induced liver injury	hUC-MSCs	EV	ultracentrifugation	/	male Sprague-Dawley	/	systemic	10 mg/kg EV	hUC-MSC-EVs ↘ histologic lesions, inflammation, neutrophil infiltration, oxydative stress, apoptosis, ALT, AST, and ALP level. hUC-MSC-Evs carry antioxidant enzyme.	Yao et al. (293), doi: 10.1096/fj.201800131RR
						vitro 1: human LO2 cells - vitro 2: neutrophils LPS activated	/	/	20 µg EV		
	I/R induced liver injury	Human-induced pluripotent stem cell derived MSC	Exosomes	ultrafiltration/ultracentrifugation.	/	male Sprague-Dawley	end of procedure	inferior veina cave	600 µg suspended in 400 µL	Evs, ↘ histological lesions, hepatic enzymes levels, oxydative stress, inflammation (infiltration cells,HMGB1, TNF-α and IL-6↘). Protect hepatocyte (apoptosis↘ proliferation↗).	Nong et al. (294) https://doi.org/10.1016/j.jcyt.2016.08.002
	I/R induced liver injury	hUC-MSCs	Exosomes	ultracentrifugation	Transfection with : miR-1246 inhibitor	C57BL/6 mice	0h after reperfusion	/	2.5 × 10'12 exosome diluted in 500 µL	hUCB-MSCs-derived exosomes ↘ apoptosis in vitro, histological lesions, enzymatic release (AST, ALT) and cytokines (TNF-α, IL-6 and IL-1β↘). via miR-1246 and GSK3β-Wnt/β-catenin pathway activation.	Xie et al. (295), https://doi.org/10.1080/15384101.2019.1689480
						vitro: LO2 cells exposed to hypoxia/reoxygenation (H/R)	coculture	/	/		
	I/R induced liver injury	hUC-MSCs	Exosomes	exosome isolation kit	/	C57BL/6 male mice	0h after reperfusion	systemic	10 μg/100 μL exosomes	hUCB-MSCs-derived exosomes ↘ histological lesions, enzymatic release and Th17/Treg ratio in CD4+ T cells in vitro, via the IL-6-gp130-STAT3 pathway	Xie et al. (296), 2019 doi: 10.1002/iub.2147
						vitro: naïve human CD4+ T cocultured with LO2 and tranfected with IL-6 signal transductor	coculture	/	/		
	I/R induced liver injury	hUC-MSCs	EV	ultracentrifugation	/	C57BL/6 male mice	0h after reperfusion	systemic	100 µg/100 µL EV	Evs ↘ inflammatory response by decrease CD154 expression on T CD4+, via CCT2 and NFAT1 signaling pathway.	Zheng et al. (297), doi: 10.1002/advs.201903746
						vitro 1: intrahepatic mononuclear cells - vitro 2: CD4+ T	/	/	/		
	I/R induced liver injury	Human bone marrow derived MSC	EV	ultracentrifugation	/	C57BL/6 female mice	5 min before procedure	systemic	1 x 10'9 EV/ 200µL	MSC-derived EV ↘ serum transaminase levels, hepatic necrosis,transcription of inflammation-associated genes, and ↗ number of Ki67-positive hepatocytes	Anger et al (298), doi: 10.1089/scd.2019.0085
KIDNEY	Glycerol induced AKI	Human bone marrow derived MSC	EV	ultracentrifugation	/	male CD1 nude mice	3 days after injury	systemic	200 μg	EVs accumulated specifically in the kidneys of the mice with AKI compared with the healthy controls	Grange et al. (299), DOI: 10.3892/ijmm.2014.1663
						vitro: Human renal proximal tubular epitheial cells	coculture with EV	/	50 μg/mL EV		
	I/R induced AKI	Human bone marrow derived MSC	MV	ultracentrifugation	/	male Sprague-Dawley rat	end of procedure	systemic	30 µg of MV	MV ↘ apoptosis, functionnal lesions, ↗ stimulating tubular epithelial cell proliferation.	Gatti et al. (300), doi: 10.1093/ndt/gfr015
	I/R induced AKI	Human amnion epithelial cell derived exosomes	Exosomes	ultracentrifugation	/	Male C57BL/6j mice	end of procedure	systemic	3 × 10'8 exosomes	Exosomes ↘histological, functionnal lesions, apotosis, ↗ cells proliferation, density of peritubular capillars, M2 polarization, and anti-inflammatory effects (IL-4, IL-13↗, TNF-α,IFN-γ ↘)	Ren et al. (301), https://doi.org/10.1186/s13287-020-01917-y
						vitro: HK-2 cells exposed to hypoxia during 48h	coculture with EV	/	1 × 10'8/ml exosomes		
	I/R induced AKI	Rat Adypocytes tissu derived MSC	Exosomes	ultracentrifugation	/	male Sprague-Dawley rat	3h after injury	systemic	exosome (100 μg)], and/or ADMSC (1.2 × 10'6 cells)	Exosomes ↘ histological, functionnal lesions, apoptosis , oxydative stress, inflammation (TNF-α, NF-κB, IL-1β, MIF, PAI-1 and COX-2 ↘at 72h )	Lin et al. (302) https://doi.org/10.1016/j.ijcard.2016.04.061
	Rhabdomyolisis via glycerol induced AKI	Human bone marrow derived MSC	MV	ultracentrifugation	Transfection with shRNAmiR targeting Drosha	SCID Mice	3 days after injury	systemic	2.2×10'8MV diluted in 150µL	miARN allow MV therapeutics effects	Collino et al. (303). doi: 10.1681/ASN.2014070710
						vitro: tubular epithelials cells, for C57BL/6 female mice	coculture with MV	/	/		
	I/R induced AKI	hUC-MSC	EV	ultracentrifugation	/	male Sprague-Dawley rat	end of procedure	systemic	100 µg of MV diluted in 0,5 mL	EV ↘ NK (and CX3CL1 - TLR2) up-regulation. EV action is allowed by carriying ARN	Zou et al. (304), doi: 10.1089/hum.2016.057
						vitro: human umbilical vein endothelial cells	coculture with EV	/	/		
	I/R induced AKI	hUC-MSC	MV	ultracentrifugation	IFN-γ during 24 or 48h	male Sprague-Dawley rat	end of procedure	systemic	/	MV without priming are better to protect kidney (Histological and functionnal lesions ↘). MV promote Treg proliferation. Priming with INFγ modulate MV material carrying and origin.	Kilpinen et al. (305), http://dx.doi.org/10.3402/jev.v2i0.21927
						vitro: PBMC	coculture with MV	/	/		
	Rhabdomyolisis via glycerol induced AKI	Human bone marrow derived MSC	EV	ultracentrifugation	/	SCID Mice	3 days after injury	systemic	165 × 10'6 particules diluted in 120µL	EV population enriched in exosomes ↘ histlogical and functionals lesions comparable with total EV population. Enriched in specific mRNAs (CCNB1, CDK8, CDC6) in comparaison with EV population enriched in MV	Bruno et al. (306), doi: 10.1089/ten.tea.2017.0069
						vitro: murine epithelials cells	coculture with particules	/	/		
	I/R induced AKI	hUC-MSC	EV	ultracentrifugation	Rnase pre treatement of EV	male Sprague-Dawley rat	end of procedure	systemic	100 µg of MV diluted in 1mL	EV ↗ renal VEGF, ↘ fibrosis and HIF-1α. Rnase treatement abrogate benefits	Zou et al. (307), doi:8(10): 4289–4299.
						vitro1: rat tubular epithelial cells	coculture with EV	/	/		

↗ mean: increase, and ↘ mean: decrease

### 5.1 MSC-EVs and Intestinal Injury

To our knowledge, no study has investigated the role of EV administration in THS-induced intestinal ischemia. Most studies have investigated the role of EVs in inflammatory bowel diseases (IBDs), mainly ulcerative colitis and Crohn’s disease. A number of rodent models of colitis have been developed; among them, chemical models, notably dextran sulfate sodium (DSS), are widely used (308). The data listed below will relate to the results obtained in this context.

The intravenous injection of MSC-EVs from different sources [bone marrow, umbilical cord, and adipose-derived stromal cells (ADSCs)] attenuated the severity of colitis. Indeed, they exert antioxidative and anti-apoptotic effects, they also reduce the mRNA and protein levels of NF-kB, numerous cytokines, chemokines (TNF-α, IFN-γ, IL-12, IL-1β, IL-6, IL-7, CCL-24, and CCL-17) and enzymes (iNOS and COX2) and they increase IL-10 and TGF-β in the injured colon (279, 280, 282). However, it was observed that TSG-6 depletion in EVs reduced their immunomodulatory efficacy. TSG-6 in EVs plays a key role in increasing the population of Tregs and for macrophage polarization from M1 to M2 in the colon (269). Moreover, intraperitoneal injection of MSC-Exo in a mouse model of inflammatory bowel disease indicated a protective role in the intestinal barrier not only by preventing the destruction of tight junctions, therefore decreasing permeability, but also by modulating the responses of Th2 and Th17 cells in the mesenteric lymph nodes. Again, the knockdown of TSG-6 abrogated the therapeutic effects of MSC-Exo; conversely, administration of a recombinant of TSG-6 showed beneficial effects similar to those of MSC-Exo (286). Therefore, TSG-6 appears to play a major role in the anti-inflammatory effects of MSC-EVs in inflammatory bowel pathologies. Moreover, another study revealed that bone marrow MSC-EVs could inhibit the differentiation of Th17 cells in ulcerative colitis by regulating histone H3 lysine-27 trimethylation (H3K27me3) that is closely associated with the differentiation of Th17 cells. Therefore, MSC-EVs, which regulate H3K27me3, could be promising agents for inflammatory immune diseases associated with abnormal Th17 cell differentiation (283). Administration of MSC-EVs also increases the percentages of CD4^+^ CD25^+^Foxp3^+^ Tregs in lymph nodes and the spleen (281).

Moreover, as is described in other pathologies, TNF-α and IFN-γ MSC priming increased the immunosuppression of MSC-EVs (270). Finally, Yu et al. evaluated the effect of EphB2-overexpressing bone marrow MSC-EVs. EphB2 is a signaling receptor involved in the regulation of inflammatory response, immune homeostasis, and cell migration. They showed that the overexpression of EphB2 improved the colonic targeting ability of EVs and demonstrated a robust immunomodulatory effect by the modulation of the Th17/Treg balance (278).

Regarding the potential beneficial effect of the miRNA content in EVs in this pathology, a crucial role of exosomal miR200b has been described by using heme oxygenase-1 (HO-1)-modified bone marrow MSC-Exo, which overexpresses miR200b. This miRNA targets the *HMGB3* gene involved in intestinal inflammation (284). Moreover, EVs overexpressing miR-146a seem to exert better anti-inflammatory effects in an experimental rat model of colitis (285). Furthermore, the study of Wang et al. demonstrated a stronger therapeutic effect of human umbilical cord (hUC)-MSC-derived exosomes that highly expressed miR-326. Indeed, this miRNA played an important role in the inhibition of the neddylation process that indirectly activated NF-κB pro-inflammatory transcription factor (277).

### 5.2 MSC-EVs and ALI or ARDS

MSC-EVs have been extensively studied in septic ALI, including in clinical trials (309) and, more recently, in COVID-19 patients (**Table 2**). In the few models of ALI induced by THS, it was demonstrated that bone marrow MSC-EVs can modulate cytoskeletal signaling and attenuate lung vascular permeability (290). Moreover, ADSC-MSC-EVs could decrease endothelial damage *via* the PI3K/Akt signaling pathway (289). In a mouse model of lung I/R injury, EV treatment significantly attenuated lung dysfunction and injury by decreasing edema, neutrophil infiltration, and myeloperoxidase levels. Moreover, significant decreases in pro-inflammatory cytokines (IL-17, TNF-α, and CXCL1) and HMGB1 were observed. An upregulation of KGF, PGE2, and IL-10 in the bronchoalveolar fluid was also shown. Finally, MSC-EVs significantly downregulated the iNKT-produced IL-17 and the macrophage-produced HMGB1 and TNF-α in an *in vitro* model of hypoxia/reoxygenation (291). Moreover, as described previously, intestinal I/R is a common clinical occurrence caused by a number of pathophysiological contexts, including THS. ALI is a primary component of MOF triggered by intestinal I/R. In a rat model of ALI induced by occlusion/reperfusion of the superior mesenteric artery, intravenous treatment by rat bone marrow MSC-derived exosomes attenuated lung damage by decreasing apoptosis and the pulmonary levels of pro-inflammatory cytokines such as TNF-α, IL-6, and IL-1β, accompanied by a downregulation of the expressions of TLR4 and NF-κB (288).

**Table 2:** Overview of the applications of mesenchymal stromal cell-derived extracellular vesicles (MSC-EVs) in clinical studies.

NCT Number	Title	Status	Conditions	Interventions	Outcome Measures	Sponsor / Collaborators	Phases	Enrollment	Study Type	Study Designs	Other IDs or DOI	Start Date	Locations	Results, if published
NCT04384445	A Phase I/II Randomized, Double Blinded, Placebo Trial to Evaluate the Safety and Potential Efficacy of Intravenous Infusion of Zofin for the Treatment of Moderate to SARS Related to COVID-19 Infection vs Placebo	Recruiting	Covid19 Corona Virus Infection SARS (Severe Acute Respiratory Syndrome) Acute Respiratory Distress Syndrome	Drug: ZofinTM Versus Placebo	Incidence of any infusion associated adverse events | Incidence of Severe Adverse Events Safety | Survival Rate|Cytokine Levels | D-dimer Levels | C-reactive protein Levels |Quantification of the COVID-19 |Improved Organ Failure | Chest Imaging Changes	Organicell Regenerative Medicine	Phase I / II	20	Interventional	Allocation: Randomized | Intervention Model: Parallel Assignment | Intervention Model Description:parallel |Masking: Double|Double blind |Primary Purpose: Treatment	19881	September 8, 2020	Larkin Community Hospital Miami,and Hospital South Miami, Florida, United States	Not Published
NCT04602104	A Multiple, Randomized, Double-blinded, Controlled Clinical Study of Allogeneic Human Mesenchymal Stem Cell Exosomes (hMSC-Exos) Nebulized Inhalation in the Treatment of Acute Respiratory Distress Syndrome	Not yet recruiting	Acute Respiratory Distress Syndrome	Biological: Exosome of MSC (High, medium or low dose)	Incidence of adverse reaction | Murray lung injury score | PaO2/FiO2 | SOFA score | ApachII score | Number of deaths | The number of days that survivors were offline for mechanical ventilation | The number of days the survivor was out of ICU | Incidence of treatment emergent adverse event	Ruijin Hospital / Cellular Biomedicine Group Ltd.	Phase I / II	169	Interventional	Allocation: Randomized |Intervention Model: Parallel Assignment |Masking: Double (Participant, Investigator) |Primary Purpose: Treatment |	MEXARDS	October 2020	Ruijin Hospital, Medical School of Shanghai Jiaotong University and Shanghai, Shanghai, China	Not Published
NCT04798716	Mesenchymal Stem Cell Exosomes for the Treatment of COVID-19 Positive Patients With Acute Respiratory Distress Syndrome and/or Novel Coronavirus Pneumonia	Not yet recruiting	Covid19 Novel Coronavirus Pneumonia Acute Respiratory Distress Syndrome	Drug: MSC-exosomes delivered intravenously every other day on an escalating dose: (2:4:8) or (8:4:8) or (8:8:8)	Measure and report treatment-related-adverse events | Quantify safety of ARDOXSO™ |Tabulate and report the number of IMV days | Analyze and report organ failure, associated with ICU mortality | Correlate and analyze the SOFA score| Record and analyze respiratory measures (Berlin Score/PEEP) | Quantify efficacy of interventional exosome therapy in COVID-19	AVEM HealthCare	Phase I / II	55	Interventional	Allocation: Randomized |Intervention Model: Sequential Assignment | Masking: Double (Participant, Care Provider) | Primary Purpose: Treatment	20582	September 2021	Mission Community Hospital Panorama City, California, United States	Not Published
NCT04493242	Bone Marrow Mesenchymal Stem Cell Derived Extracellular Vesicles Infusion Treatment for COVID-19 Associated Acute Respiratory Distress Syndrome (ARDS): A Phase II Clinical Trial	completed	Covid19 ARDS Pneumonia, Viral	Biological: DB-001 Versus Placebo	PaO2/FiO2 ratio | Time to Recovery | Incidence of Serious Adverse Events | All-cause Mortality | (SARS-CoV-2) Ribonucleic Acid (RNA) Level | Viremia | CRP, D-dimer, Ferritin, IL-6, TNF-α | Immune Cell Counts |SOFA scoring |Standardized Quality of Life Metric	Direct Biologics, LLC	Phase II	120	Interventional	Allocation: Randomized | Intervention Model: Parallel Assignment | Masking: Triple (Participant, Care Provider, Investigator) | Double-blinded | Primary Purpose: Treatment	DB-001 // doi: 10.1089/scd.2020.0080. Epub 2020 May 12.	September 24, 2020	Helen Keller Hospital Sheffield, Alabama, United States, 35660| St. Joseph Hospital Heritage Fullerton, California, United States, 92835 | Donald Guthrie Foundation/ Robert Packer Hospital Sayre, Pennsylvania, United States, 18810 | Covenant Health Lubbock, Texas, United States, 79410 | PRX Research Mesquite, Texas, United States, 75149	Safty profile | Restore oxygenation | Downregulate cytokine storm |Reconstitute immunity
NCT04276987	A Pilot Clinical Study on Aerosol Inhalation of the Exosomes Derived From Allogenic Adipose Mesenchymal Stem Cells in the Treatment of Severe Patients With Novel Coronavirus Pneumonia	Completed	Coronavirus	Biological: MSCs-derived exosomes	Adverse reaction and severe adverse reaction |Time to clinical improvement |Number of patients weaning from mechanical ventilation | Duration (days) of ICU monitoring | Duration (days) of vasoactive agents usage | Duration (days) of mechanical ventilation supply | Number of patients with improved organ failure | Rate of mortality | (SOFA) score | Biologicals measure	Ruijin Hospital / Shanghai Public Health Clinical Center Wuhan Jinyintan Hospital, Wuhan, China Cellular Biomedicine Group Ltd.	Phase I	24	Interventional	Allocation: N/A | Intervention Model: Single Group Assignment | Masking: None (Open Label) | Primary Purpose: Treatment	MEXCOVID	February 15, 2020	Ruijin Hospital Shanghai Jiao Tong University School of Medicine Shanghai, Shanghai, China	Not Published
NCT04602442	The Extended Protocol of Evaluation of Safety and Efficiency of Method of Exosome Inhalation in COVID-19 Associated Two-Sided Pneumonia	Enrolling by invitation	Covid19 SARS-CoV-2 PNEUMONIA COVID-19	Drug: EXO 1 inhalation Drug: EXO 2 inhalation Drug: Placebo inhalation	Number of participants with non-serious and serious adverse events during trial |Time to clinical recovery |SpO2 concentration changes | Chest Imaging Changes | CRP |Procalcitonin concentration | Ferritin concentration | Creatinine concentration |Urea concentration	Clinics of the Federal State Budgetary Educational Institution SSMU Samara Regional Clinical Hospital V.D. Seredavin	Phase II	90	Interventional	Allocation: Randomized | Intervention Model: Parallel Assignment| Masking: Double (Participant, Care Provider) |Primary Purpose: Other	COVID-19 EXO Extended	October 1, 2020	Medical Centre Dinasty Samara, Russian Federation	Not Published
NCT04356300	Exosome of Mesenchymal Stem Cells for Multiple Organ Dysfuntion Syndrome After Surgical Repaire of Acute Type A Aortic Dissection	Not yet recruiting	Multiple Organ Failure	Biological: Exosome of MSC	survival after intervention|sequential organ failure assessment score|interleukin-6|The number of allergic reactions|The number of people who get cancer|the effects on kidney function|the effects on liver function|the effects on lung function|the effects on coagulation function|the effects on central nervous system	Fujian Medical University	Not Applicable	60	Interventional	Allocation: Randomized | Intervention Model: Parallel Assignment|Masking: Single (Outcomes Assessor)|Primary Purpose: Treatment	2020005	September 1, 2020	Fujian Medical University, Fujian Province, China	Not Published
**Reaserch terms in Clinical Trials (https://clinicaltrials.gov/ct2/home) on August 25th 2021**														
**Statues**	**Conditions or disease**			**Other Terms**										
All studies	lung disease			microvesicules										
	lung dysfunction			exosomes										
	lung injury			microparticules										
	acute lung injury			extracellular vesicules										
	acute respiratory distress syndrom			exosomes of mesemchymal cells										
	kidney disease			MSC derived										
	kidney injury													
	kidney dysfunction													
	acute kidney injury													
	liver disease													
	liver injury													
	liver dysfunction													
	acute liver injury													
	gut disease													
	gut injury													
	gut dysfunction													
	bowell disease													
	bowell injury													
	bowell dysfunction													
	inflammation													
	ischemia reperfusion injury													
	multiple organ failure													
	multiple organ dysfunction													
	multiple trauma													
	blunt trauma													
	blunt injury													
	haemorrhagic shock													
	traumatic hemorrhage													
	traumatic hemorrhage shock													
	war injury													
	war related trauma													
	war related injuries													

EV miRNAs also play a role. miR-124-3p, abundantly expressed in rat MSC-derived exosomes, inhibits the expression of the purinergic receptor P2X ligand gated ion channel 7 (P2X7). Inhibition of P2X7, which is overexpressed in traumatic ALI rats, improves oxidative stress and decreases the levels of inflammatory factors, including TNF-α, IL-6, and IL-8, and increases IL-10 (287). Furthermore, the transfer of MSC-EV miR-451 to macrophages *in vitro* not only inhibits TNF-α and macrophage migration inhibitory factor secretion but also represses their TLR signaling. This repression allowed the mitochondrial transfer of MSC-EVs to macrophages. All these immunomodulatory effects on macrophages were exerted by different MSC-EV populations (310). Altogether, these data indicate that MSC-EVs, by limiting oxidative stress and vascular permeability and by downregulating the activity of immune cells in the lungs, represent a novel therapeutic option in the treatment of traumatic ALI.

### 5.3 MSC-EVs and Acute Liver Injury

As for ALI, many studies have evaluated MSC-EVs in models of hepatic injury induced by the administration of d-galactosamine/TNF-α, various toxic drugs, or LPS. Systemic administration of human MSC-EVs on hepatic I/R injury suppressed not only hepatocyte necrosis and sinusoidal congestion but also AST and ALT injury markers (294, 298, 311). Moreover, in a model of I/R-induced hepatic apoptosis, hUC-MSC-EVs reduced neutrophil infiltration and, therefore, their respiratory burst. This alleviated oxidative stress in hepatic tissue (293). This suggests that MSC-EVs could reduce hepatic injury by suppressing inflammatory responses (of TNF-α, IL-6, and HMGB1) and attenuating the oxidative stress response [by increasing glutathione, glutathione peroxidase, and superoxide dismutase (SOD)] and apoptosis (by decreasing caspase-3 and Bax) (292, 294). hUC-MSC-EVs could induce anti-apoptotic and pro-survival effects in a human liver cell line and ameliorated the I/R injury-induced hepatic dysfunction in mice. This study highlighted the crucial role of miR-1246 *via* the regulation of the GSK3β-Wnt/β-catenin pathway to mediate these effects (295). Subsequently, exosomes expressing miR-1246 had protective effects against hepatic I/R by regulating Th17/Treg imbalance *via* the interaction of miR-1246 and IL-6-gp130-STAT3 (296). Another team described in an I/R mouse model that hUC-MSC-EVs significantly modulated the membranous expression of CD154 of intra-hepatic CD4^+^ T cells, which initiated the inflammatory response in the liver and can aggravate liver I/R (297). As shown in the few studies exploring the effects of treatment with MSC-EVs after I/R, their capacity to inhibit immune cell activation (mainly neutrophils) and pro-inflammatory cytokine release, as well as their capacity to attenuate oxidative stress and to inhibit hepatic cell apoptosis, makes MSC-EVs a promising therapy to treat liver injury following THS.

### 5.4 MSC-EVs and AKI

Many studies have shown the beneficial effects of the administration of MSC-EVs in AKI (312). As in the previous sections, only studies using models of I/R or rhabdomyolysis were discussed since toxicity studies (cisplatin) are not relevant to THS. The therapeutic effects of EVs are mediated by different biological processes, including anti-apoptosis, anti-inflammation, angiogenesis, and anti-fibrosis (303, 306, 307). After systemic injection, labeled MSC-EVs accumulated specifically in the kidneys of mice with AKI, but not in healthy controls (299). This suggests a homing capacity of EV-derived MSCs on the site of injury.

In an I/R-induced AKI mouse model, exosomes from human amnion epithelial cells (hAEC-Exo) could improve animal survival and renal function and induce M2 macrophage polarization. This M1/M2 shift was associated with increases in the IL-4 and IL-13 levels and decreases in the TNF-α and IFN-γ levels, which helped reduce the inflammatory response (301). Similarly, EVs from ADSCs decreased the protein levels of NF-κB, TNF-α, IL-1β, and MIF, as well as PAI-1 and COX-2 in the kidney parenchyma, 72 h after I/R (302). Moreover, administration of human Wharton jelly MSC-EVs also alleviated inflammation (decreased TNF-α and increased IL-10 expressions in the kidney) in the first 48 h, but also suppressed the expression of CX3CL1 (a potent chemo-attractant factor for macrophages) and decreased the number of CD68^+^ macrophages in the kidney (231). Several studies suggest that the therapeutic effects of EVs can be mediated by functional mRNAs and miRNAs (228, 300, 303). MSC-EVs express high levels of miR-15a, miR-15b, and miR-16 that may modulate CX3CL1 expression (231). The same team also described that the number of NK cells increased in the kidney after I/R injury. EVs also decreased the percentage of NK cells in ischemic kidney, suggesting that MSC-EVs could alleviate kidney injury by regulating NK cells (304). Several proteins expressed by both naive and IFN-γ-primed EV-MSCs, such as galectin-1 and galectin-3 described as mediators of MSC T-cell immunosuppression, or the membrane markers CD90 and CD73 are also associated with MSC-immunosuppressive capacity (305). Finally, EV-MSCs contain anti-inflammatory and anti-oxidative apolipoprotein A1 (ApoA1). ApoA1 is described to have therapeutic effects in kidney injury, leading to the reduction of serum creatinine levels, serum TNF-α and IL-1β levels, and tissue MPO activity. Moreover, ApoA1 can suppress the expressions of ICAM-1 and P-selectin in the endothelium, thus diminishing neutrophil adherence (313). This literature, reduced here to I/R and rhabdomyolysis injuries, indicates the benefit of treatment with MSC-EVs of AKI by limiting the leukocyte chemoattraction and activation through inducing a shift from M1 to M2 macrophages or by decreasing pro-inflammatory and increasing anti-inflammatory cytokine production. All these encouraging arguments suggest that there is a potential interest in the use of MSC-EVs in the context of THS.

### 5.5 MSC-EVs: A New Hope for the Prevention of MOF?

The pathophysiology of THS-induced MOF is complex and still not fully understood. The aim of most treatments currently used in the clinic is to compensate for the function of the affected organ with, for example, dialysis, parenteral nutrition, or controlled ventilation and oxygenation. Limited options are available to prevent the occurrence or limit the extent of organ failure in THS. The imbalance between SIRS and CARS is a key mechanism in MOF, but because it is at the crossroad of multiple system dysfunctions, no unique physiological or molecular therapeutic target can be identified. As shown in previous sections, MSCs and their EVs have an important potential to treat isolated organ failure through multiple intricate molecular mechanisms that target notably inflammation and oxidative stress. This is the reason why we believe that taking advantage of the pleiotropic effects of MSC-EVs could be a precious new approach in a pathophysiological situation as complex and multifactorial as that of THS leading to MOF.

## 6 MSC-EVs: Toward a Clinical Grade Production

MSC-EVs represent a great hope for the treatment of THS. Their use can have important advantages, but unknowns persist. Although a number of preclinical studies have explored the biology of MSC-EVs, only a few clinical trials have been listed concerning acute injuries of isolated organs, systemic immune dysfunctions, I/R injuries, or trauma and MOF (**Table 2**). A significant increase has occurred with the SARS-CoV-2 pandemic, and complete studies indicated that the administration of MSC-EVs decreases systemic inflammation and allows restoration of pulmonary oxygenation; most other studies are in progress.

Regarding the systemic administration of EVs, which seems the most relevant in the context of THS-induced MOF, although the biodistribution/homing of MSCs has been explored, it is still poorly understood for EVs. However, it was demonstrated that 70% of near-infrared lipophilic dye-labeled human MSC-EVs accumulated in the liver after systemic administration in healthy mice. Interestingly, dendritic cell-derived EVs showed an increased accumulation in the spleen, suggesting that the homing pattern of EVs reflects those of their original cells (314). On the other hand, the unpredictable nature of THS, as well as the need for emergency administration of the therapeutic product, requires the use of EVs from allogeneic MSCs. It is known that the survival of allogeneic MSCs is limited after administration, but during this time, they continuously secrete soluble factors/EVs, adapted to the pathophysiological context that they encounter. This is not the case with EVs, but iterative administrations can be more easily considered. Indeed, it will be a product already prepared/qualified, immediately available, and easily stored and transportable, allowing patients to be treated anywhere without the need for nearby production facilities. In addition, we hope that the lack of adaptability of EVs to the pathophysiological context could be compensated by the use of optimized priming upstream.

**Figure 4:**
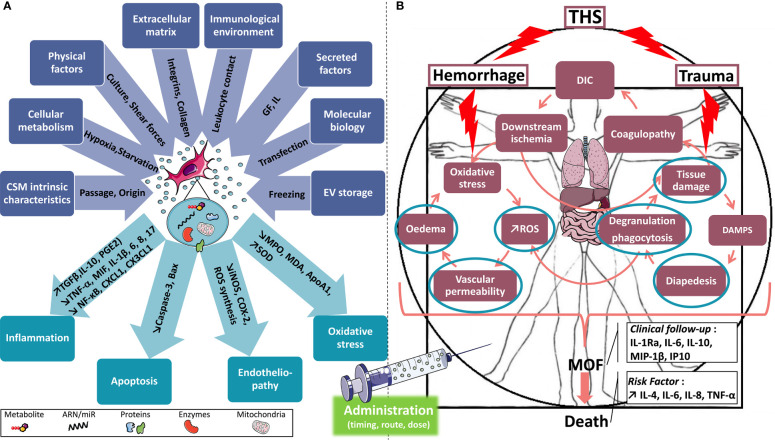
MSC-EVs in THS, or how to apply recent knowledge at the service of those seriously injured. **(A)** In *blue* are the main factors modulating the production of EVs. EVs in the *center* contain, depending on the priming, organelles, proteins, enzymes, RNA, and miR in variable quantities. In *aquamarine* are the possible main pathways of the potential beneficial effects of EVs in THS models. The administration methods vary by model and must be explored. **(B)** Simplified consequences of THS. Three loops (coagulopathy, inflammation, and endotheliopathy) are involved in the vicious circle leading to MOF. Cytokines of clinical interest are predictors of the onset of MOF. IL-4, IL-6, IL-8, and TNF-α are significantly increased in trauma patients with MOF and not surviving it. Items circled in aquamarine are potential targets for EV action. EVs, extracellular vesicles; MSC-EVs, mesenchymal stromal cell-derived extracellular vesicles; THS, traumatic hemorrhagic shock; MOF, multi-organ failure; MPO, myeloperoxidase; MDA, malondialdehyde; SOD, superoxide dismutase; GF, growth factor; IL, interleukin.

In addition, as has been the case with MSCs, there may be a mismatch between the hope raised by exciting preclinical publications and the ability to enter daily clinical practice. These difficulties could result not only from differences between human and animal species but also from the heterogeneity of the products used. Variability in MSC-EVs is associated with the variability of the cells from which they are produced. The variability of MSCs arises from several key factors such as the tissue origin (bone marrow, adipose tissue, perinatal tissues, etc.), donor, culture condition media/support (platelet lysate, fetal bovine serum, bioreactor, priming by hypoxia, or cytokines), age (age of donor and culture passage), or cryopreservation. Moreover, depending on the therapeutic target, a strategic choice between primary MSCs or cell line, native or modified, must be carefully considered. Therefore, EVs could be selected based on the advantages of MSC sourcing/efficacy. A study comparing the protein profiles of MSC-EVs with the proteome profiles of EVs from other cells showed a specific protein signature of MSC-EVs, despite the huge diversity in the sources of MSCs or the preparation methods of MSC-EVs. However, 22 proteins were exclusively found in the bone marrow-derived MSC-EV profiles. Identification of the functional markers of potency and the development of easily deployable and standardized methods of evaluation would benefit the field of EVs, as it did for cell therapy, and in this study, it was also suggested that several membrane and extracellular proteins (i.e., COL6A2 or COL6A3 and THY1) could be used as a standard for the quality control of production either in research or in clinical settings (259). EVs can also be transformed/loaded (without prior transformation of the producing MSCs) to improve their targeting or their therapeutic properties. All these provide a large field of possibilities for the clinical use of EVs (315). Moreover, in most EV manufacturing processes, the therapeutic product is composed of a continuum of different types of vesicles (size and origin) and certain amounts of soluble proteins that may participate in the biological and therapeutic activity of the final product (**Figure 4**). In fact, most of the studies described in the literature are based on products that do not consist solely of EVs (due very often to isolation by ultracentrifugation), but which contain a greater or a lesser proportion of soluble proteins. The most effective product could therefore be an “EV-enriched secretome.” In preclinical studies, EVs are isolated using different techniques: ultracentrifugation, tangential filtration, immunocapture, or precipitation (252). However, not all of them are easy to consider when moving to clinical grade production. Indeed, although ultracentrifugation is the most widely used, it is time-consuming and additional stages of purification (washing and microfiltration) are generally necessary to increase the purity of the EV products. Tangential flow filtration, for example, already validated for industrial-scale productions, seems more suitable. On the other hand, specifically concerning MSCs, the culture media used for the expansion phases are enriched with fetal calf serum or platelet lysate, which contain large amounts of EVs that cannot be distinguished or separated from MSC-derived EVs. To overcome this problem, in many studies, the culture medium is removed after the expansion of MSCs, rinsed, and replaced by a medium without these additives during the entire period of MSC secretion. The cellular stress generated by starving must, however, be taken into consideration since it generates modifications in the state of the cells and, therefore, in the quality/functionality of the EVs produced. On the other hand, the secretion times of the EVs, and therefore the potential quantity of EVs recovered, are limited. The alternatives for the clinic consist in the use of serum-free or platelet lysate-free media (containing specific cocktails of growth factors and additives), but these media are very specific of a cell type and very expensive, which is problematic for the large-scale production of conditioned medium. Commercial “exosome-free” media also exist. Depletions are performed by ultracentrifugation; however, the levels of depletion are not optimal, with variations depending on the centrifugation conditions and durations. Likewise, tangential flow filtration appears to be a possible solution concerning the purification of culture media for large-scale clinical productions.

Finally, from a regulatory point of view, EV-derived products are classified as medicinal products. Within the framework of medicinal products, EV-derived products are categorized as “biological medicinal products” (Directive 2003/63/EC of June 25, 2003, amending Directive 2001/83/EC). However, MSC-EVs could be subcategorized. When they originate from unmodified primary cells or from genetically modified cells that do not contain a transgene product (immortalized cells), they belong to the biological medicinal product category, without any further subcategory. In contrast, MSC-EVs containing a transgene considered as a gene therapy product (e.g., recombinant mRNA and miRNA) are classified as gene therapy products, a subclass of advanced therapy medicinal products (ATMP). This means that the active substance and mode of action of MSC-EVs are decisive for their regulatory classification and can have significant repercussions on the manufacturing process. The use of primary MSCs may have some limitations for large clinical-scale manufacturing due to their limited life span and the donor-to-donor or batch-to-batch heterogeneity. Therefore, EVs produced from immortalized MSCs could be the most promising strategy to prevent MOF.

## 7 Conclusion/Discussion

In recent years, a considerable number of studies have contributed to a better understanding of the biology of EVs and paved the way for their therapeutic use. In cases of isolated organ injuries, MSC-EVs can help restore local homeostasis by decreasing inflammation and oxidative stress, by having an anti-apoptotic effect, or even inhibiting endotheliopathy. Locally protecting the onset of organ damage is a means to prevent the onset of SIRS and the depression of CARS at the systemic level, which promote the development MOF.

This new therapeutic tool could revolutionize the field of cell therapy because it opens the way to treatments that can be administered as early as possible for the care of patients, not only in civilian life but also in hostile contexts such as those encountered in theaters of military operations.

## Author Contributions

GV and JP wrote the manuscript with input from all authors. All authors (NL, CM, EV, and SB) provided critical feedback and helped shape the manuscript. All authors contributed to the article and approved the submitted version.

## Conflict of Interest

The authors declare that the research was conducted in the absence of any commercial or financial relationships that could be construed as a potential conflict of interest.

## Publisher’s Note

All claims expressed in this article are solely those of the authors and do not necessarily represent those of their affiliated organizations, or those of the publisher, the editors and the reviewers. Any product that may be evaluated in this article, or claim that may be made by its manufacturer, is not guaranteed or endorsed by the publisher.
